# Determination of Amino Acids’ p*K*_a_: Importance of Cavity Scaling within Implicit Solvation Models and Choice of DFT Functionals

**DOI:** 10.1021/acs.jpcb.3c07007

**Published:** 2024-02-12

**Authors:** Filip Šebesta, Žofie Sovová, Jaroslav V. Burda

**Affiliations:** Department of Chemical Physics and Optics, Faculty of Mathematics and Physics, Charles University, Ke Karlovu 3, 121 16 Prague 2, Czech Republic

## Abstract

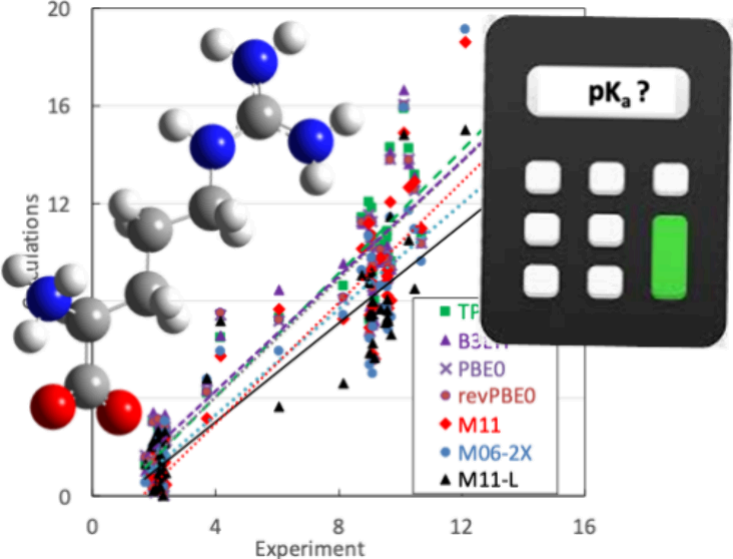

Protonation states of molecules significantly influence the thermodynamics and kinetics of chemical reactions. This is especially important in biochemical processes, where appropriate protonation states of amino acids control the exo/endoergicity of practically all biochemical cycles. This paper is focused on appraisal of the impact of DFT functionals and PCM solvation models on the accuracy of p*K*_a_ evaluations for all proteinogenic amino acids. Eight functionals (B3LYP, PBE0, revPBE0, M06-2X, M11, M11-L, TPSSh, and ωB97X-D) and four basis sets are considered, together with four kinds of implicit solvation models when additional attention is paid to a cavity construction. An influence of nonelectrostatic contributions and Wertz’s corrections on Gibbs free energy is investigated together with accuracy of provided proton solvation energy. The best model is based on the M06-2*X*/6-311++G**/D-PCM/UAKS computational level. The fitting procedure is utilized to improve the accuracy of the evaluated models. All of these results are also compared with values obtained from the COSMOtherm program and CCSD(T) calculations. Results for cysteine and histidine are discussed individually, as they can be found in different protonation states at neutral pH.

## Introduction

Proton abstraction (PA) represents one of the most abundant and most important chemical processes in nature. It is substantially influenced by the environment, in which PA takes place. Acido-basic equilibrium is generally characterized by p*K*_a_, which is commonly collected in many chemico-physical tables like the Handbook of Chemistry and Physics from CRC Press (CRC).^1^ IUPAC defines p*K*_a_ as the negative decimal logarithm of the equilibrium constant *K*_a_ of the deprotonation reaction:
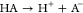
1

Since p*K*_a_ cannot always be determined experimentally, e.g., for systems that are too complex like proteins or peptides with many side chains, structures that have not been synthesized yet, some compounds that are dangerous to handle, or metastable species within a reaction mechanism, deep attention is paid to the development of reliable computational methods for p*K*_a_ determination.^2,3^ They are mostly based on evaluation of the standard Gibbs free energy (Δ*G*^0^) for the deprotonation reaction of a chosen compound according to eq 1 that is subsequently related to p*K*_a_ using the following relation:
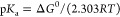
2

In eq 2, the Δ*G*^0^ energy can be obtained from calculation by treating the molecule either in the gas phase^4^ (then, gas-phase acidity is obtained, not p*K*_a_) or in solution^5,6^ or by combining these two types of calculations in a thermodynamic cycle.^7,8^ The thermodynamic cycle was proposed to benefit from both gas-phase and implicit solvation models for more accurate energy determination.^9^

Various chemical systems were suggested for p*K*_a_ calculations.^10^Reaction 1 is widely used, although its applicability is limited since it is not possible to estimate proton solvation free energy by means of quantum chemical calculations, and therefore, it must be taken from various experiments or estimated from fitting procedure based on calculated properties to the known p*K*_a_ values.^11−15^ Several approaches to proton solvation energy have been reported.^16^ One of the most generally accepted values is based on the measurement of Tissandier et al.^17^ from cluster-ion solvation data determining Δ*G*_solv_^0^(H^+^) = −265.9 kcal·mol^–1^.

In order to avoid the necessity to consider the experimental proton solvation free energy, an alternative way for the p*K*_a_ calculations can be regarded as follows:^18−20^
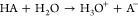
3

Using eq 3, there are some discussions in the literature whether the correction term for bulk water −log[H_2_O] = −1.74 should be used^21,22^ or not.^12,19^ For reactions where water is present as a coreactant, it is necessary to evaluate p*K*_a_ according to reaction 3 in order to get correct results.^10,23^

Another scheme on how to compute p*K*_a_ is connected with the concept of an isodesmic reaction^24^
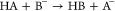
4where B stands for a reference molecule with known p*K*_a_. Then, the p*K*_a_ value of a molecule A can be computed according to the following formula:
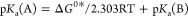
5where Δ*G*^0*^ corresponds to the standard Gibbs free energy of the reaction (eq 4). The choice of the reference molecule is crucial for an accurate p*K*_a_ determination of the molecule A. In the case of similar molecules A and B, the p*K*_a_ constant of the molecule A can be obtained with the accuracy of ca. 0.1 log unit, while for molecules with a different character, it may vary by more than 10 log units.^25,26^ In the study of Ho,^27^ it was demonstrated that direct calculation in the SMD model reproduces experimental values of p*K*_a_ in better agreement than using the thermodynamic cycles based on the reaction (eq 1) for a set of eight amino acids. Concerning the issue of accurate determination of Δ*G*^0*^ in an implicit solvent, the same authors^10^ claim that free energies obtained with the implicit solvent should be treated with thermal corrections evaluated in the gas phase. Since this argument is not generally accepted,^28^ we do not use the gas-phase thermal corrections in our implicit solvation models.

To the best of our knowledge, there are only a few systematic reports concerning p*K*_a_ calculations of free amino acids. In the group of Muñoz and Frau,^5,26^ calculations of p*K*_a_ of all proteinogenic amino acids were reported via isodesmic reactions where experimental p*K*_a_ values of alanine were used as reference for p*K*_a_ calculations of 1-carboxyl and α-amino groups. They also utilized isodesmic reactions for determination of side-chain p*K*_a_ values in small peptides using experimental p*K*_a_’s of isolated amino acids as the reference molecules. In this way, they reached an accuracy of 0.5 to 1.0 log unit. The influence of different conformations of *N*-formyl-l-histidinamide on its final p*K*_a_ was examined by Hudáky and Perczel,^29^ showing that theoretical calculations based on the fitting procedure can provide quite accurate results. High correlation between experimental and calculated p*K*_a_ values for the testing set of N-hetrocyclic molecules is the key for the fitting. A study on p*K*_a_ of cysteine focusing on the thiol group was published by Canle et al.^30^ Testing several models, namely, microsolvation, the Onsager model, the polarizable continuum model (PCM), and a hybrid model combining PCM with microsolvation, led to the conclusion that the PCM model provides the most uniform results for the thiol group. Another comparison of the hybrid model was reported by Gupta et al.^20^ when p*K*_a_’s of 10 amino acids including some nonproteinogenic molecules were explored. The RMSDs for the used PCM model were higher than 5.3 log units for 1-carboxyl and α-amino groups and higher than 2.7 log units in the case of the hybrid model. Based on these studies, it can be concluded that the fitting procedure and isodesmic reactions lead to very accurate p*K*_a_ values for similar compounds, but an appropriate reference is necessary. Microsolvation can also reduce deviations of the models; however, there is always a problem with the number of needed water molecules and their configuration. The pure PCM model is simple and more universal in this sense, but the question is how accurate it can be after some tuning (fitting) treatment.

Previously, we evaluated p*K*_a_ constants of Cys, Met, and Gly^6,12−14^ exploring several computational models, varying PCM approaches, strategies for cavity construction, basis sets, and quantum chemical methods. In this study, we focused on the p*K*_a_ determination of all the most common (proteinogenic) amino acids using eight different DFT functionals combined with 16 settings of solvation models. These results further undergo the fitting procedure for achieving higher accuracy and are compared with p*K*_a_ values obtained by Klamt’s COSMO-RS method.^2^

## Computational Methods

Optimizations of various conformations were performed at the given DFT level of theory in the Gaussian 09 program within the “Integral Equation Formalism-Polarizable Continuum Model” (IEF-PCM) framework. Eight widely used functionals were chosen: a 3-parameter Becke hybrid functional B3LYP,^31^ from the Head-Gordon laboratory a range-separated functional ωB97X-D,^32^ Perdew–Burke–Ernzerhof’s hybrid functionals PBE0^33^ and revPBE0,^62,34^ Tao–Perdew–Staroverov–Scuseria’s TPSSh,^35^ and functionals from the Truhlar laboratory, the hybrid M06-2X^36^ and next-generation pure functionals M11-L^37^ and hybrid, range-separated M11.^38^ Since the formulas for translational and rotational degrees of freedom were obtained considering ideal gas assumption, a liquid solution represents a much denser environment, and therefore, some reduction of thermal energy for these two components (translation and rotation) needs to be introduced. Based on the Wertz’s analysis, the entropy contributions *S*_trans_ and *S*_rot_ should be reduced in solution compared to the gas phase. The suggested scaling of entropy is performed according to the following formula:^39^
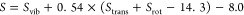
6

In subsequent single-point (SP) energy calculations, each of the considered functionals was combined with 16 models for the evaluation of solvation effects using several PCM techniques. All settings of the models are collected in Table 1. Here, we combined (a) IEF-PCM,^40^ dielectric-PCM (D-PCM),^41,42^ Cosmo-PCM or conductor-PCM (C-PCM),^41,43^ conductor-like screening model for realistic solvents (COSMO-RS),^2^ and solvation model based on solute electron density (SMD)^44^ solvent methods with (b) types of escaped charge compensations and (c) various cavity constructions. As to the last point, we evaluated radii of the spheres around the atoms using four construction schemes: vdW (universal force field radii scaled by alpha 1.1-UFF), Klamt, the united atom topological model applied on radii optimized for the PBE0 (UAKS),^45^ and scaled UAKS (s-UAKS).^12^ The different electrostatic scaling factors α (1.0, 1.1, or 1.2), and also, two strategies for smoothing of the cavity surface (vdW or SES (solvent-excluded surface)) were examined. Regarding the most important differences between various PCM/SMD methods, brief characterization is included in the Supporting Information (SI). The nonelectrostatic contributions of solvation free energy, cavitation, dispersion, and repulsion terms were also considered. These results are compared with p*K*_a_ values determined using Klamt’s COSMOtherm standalone program^46^ for obtaining the COSMO-RS results. Its input files were generated within model 15 performed by the G09 program. In addition to variation of the solvation models, the impact of the size of basis sets was also examined employing Pople’s basis sets: 6-31+G* (hereafter labeled as B1), 6-31++G** (B2), 6-311++G** (B3), and 6-311++G(2df,2pd) (B4).

**Table 1:** Settings for the Individual Solvation Models: The Type of Solvation Model, Sphere Radii for Cavity Construction, Electrostatic Scaling Factor α, Type of Cavity Surface (Smoothing), and Escaped Charge Compensation^a^

**model**	**opt**	**basis set**	**PCM**	**radii**	α	**surface**	**G03 defaults**	**charge compensation**
DFT calculations								
1	Y	B1	IEF-PCM	UFF	1.1	vdW	N	N
2	n	B2	IEF-PCM	UAKS	1.2	SES	Y	N
3	n	B2	IEF-PCM	UAKS	1.1	vdW	Y	N
4	n	B4	IEF-PCM	UAKS	1.2	SES	Y	N
5	n	B4	IEF-PCM	UAKS	1.1	vdW	Y	N
6	n	B2	D-PCM	s-UAKS	1.2	SES	Y	4
7	n	B2	D-PCM	UAKS	1.2	SES	Y	4
8	n	B3	D-PCM	s-UAKS	1.2	SES	Y	4
9	n	B3	D-PCM	UAKS	1.2	SES	Y	4
10	n	B4	D-PCM	s-UAKS	1.2	SES	Y	4
11	n	B4	D-PCM	s-UAKS	1.2	SES	Y	N
12	n	B4	D-PCM	UAKS	1.2	SES	Y	4
13	n	B4	SMD	SMD-Coul.	1.0	vdW	N	N
14	n	B4	C-PCM	Klamt	1.1	vdW	Y	N
15	n	B4	C-PCM	Klamt	1.0	vdW	N	3
16	n	B4	COSMO-RS					
CCSD(T) calculations								
CC_1	n	B3	IEF-PCM	s-UAKS	1.2	SES	Y	N
CC_2	n	B3	D-PCM	s-UAKS	1.2	SES	Y	4
CC_3	n	B2	D-PCM	s-UAKS	1.2	SES	Y	4

a The charge compensation is none (N), the electronic density weighted correction (3; ICOMP = 3 in the Gaussian input files), or the effective charge method for D-PCM (4; ICOMP = 4). A brief description of these techniques is provided in the SI. Used basis sets are 6-31+G* (hereafter labeled as B1), 6-31++G** (B2), 6-311++G** (B3), and 6-311++G(2df,2pd) (B4). “G03 defaults” means whether the older settings were applied in calculations (keyword G03Defaults).

The s-UAKS cavities^12^ represent in this case an important improvement of the standard UAKS cavity model^42,45,47^ where the radii *R*_X_ of spheres around atoms (like oxygen, phosphorus, sulfur, and chlorine) are scaled based on the natural bond analysis (NBO). Radii are linearly scaled using partial charge  of the actual atomic groups X in examined molecules vs negatively charged groups  (deprotonated) in reference molecules (or anions) in relation to the NBO charge of the protonated form of the reference molecule .^12,14,15,48,49^ The modified radii are evaluated according to the following formula:
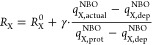
7where the scaling factors γ and the radii *R*_X_^0^ from the original UAKS model are utilized together with the  atomic charges determined from NPA (natural population analysis).^50^ In this case, the linear scaling with respect to  is smooth and leads to more consistent results. Molecules chosen for determination of the reference charges on the given groups X in the deprotonated  and protonated state  were taken as suggested in the original UAKS approach.

For additional comparison, the CCSD(T) calculations were carried out with three different settings labeled CC_1, CC_2, and CC_3 (cf. Table 1). All of them utilized the s-UAKS model for cavity construction, CC_1 with the IEF-PCM and B3 basis set and the others combining the D-PCM implicit solvation model with B3 (CC_2) and B2 basis sets (CC_3). Several other post-HF energies MP2, MP3, MP4SDQ, and CCSD obtained within these computational schemes were analyzed, too.

Inclusion of Grimme’s empirical dispersion corrections^51^ generally improves accuracy of DFT functionals. Among the used functionals, empirical dispersion is implicitly involved only in ωB97X-D. In order to reveal the importance of empirical dispersion, another set of p*K*_a_ calculations was performed with Grimme’s D3 dispersion corrections and Becke–Johnson damping^52^ in the case of the B3LYP functional for all solvation models. Nevertheless, the dispersion corrections had no substantial effect on p*K*_a_ evaluation, and therefore, we omitted these results from further discussion.

The p*K*_a_ values were (directly) determined using eq 3. Nevertheless, for higher accuracy, the least-squares fitting procedure was also introduced, and p*K*_a_ values were determined according to eq 1 where the unknown Δ*G*_solv_(H^+^) free solvation energy of H^+^ (parameter *b*) is obtained from a fit of the relation:
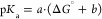
8toward experimental p*K*_a_ values of all amino acids. The Δ*G*^0^ energy represents the difference of Gibbs free energies of pertinent protonated and deprotonated forms of the given amino acid. The factor *a* = 0.733 kcal^–1^·mol corresponds to 1/(2.303*RT*) at 298.15 K. However, the parameter *b* covers not only Δ*G*_solv_(H^+^) but also corrections for −log[H_2_O]/*a* (2.38 kcal·mol^–1^), translational motion of protons (6.28 kcal·mol^–1^), and the change of the concentration to 1 mol·dm^–3^ from ideal gas (−1.89 kcal·mol^–1^). The extracted proton solvation free energies (Δ*G*_solv_(H^+^)) from the fitted parameter *b* can be compared with the experimental value of −265.9 kcal·mol^–1^.^17^

For comparison with experimental data, values from the CRC tables^1^ are used. In the case of Arg, other available experimental p*K*_a_’s^53−55^ are discussed as well.

## Results and Discussion

### Ionic Product of Water

Reproduction of the ionic product of water, p*K*_w_ = [H_3_O^+^][OH^–^], is an important test for accurate p*K*_a_ determination because Gibbs free energies of water and hydronium are required in all p*K*_a_ calculations. From this point of view, model 6 represents the best choice for all functionals in order to reproduce the experimental p*K*_w_ value, cf. Table 2. It provides p*K*_w_’s in the range of 13.4 (M11) to 17.2 (M11-L). For comparison, Table 2 also contains p*K*_w_ values obtained with model 8, the most accurate model for reproduction of the p*K*_a_ constants of all amino acids, and model 10. These three models 6, 8, and 10 differ only in the size of the used basis set: B2, B3, and B4, respectively. All the remaining settings of the solvation models are entirely the same. It reveals a trend that the value of p*K*_w_ increases with a larger basis set within this solvation model, which shifts the calculated values away from the experimental one (except the M11 functional). This trend can be explained by the fact that the UAKS model was developed and fitted with the 6-31G(d) basis set or with 6-31+G(d) for anions. Therefore, the more extended basis set does not bring expected improvements of p*K*_a_/p*K*_w_ values. In the last line of Table 2, p*K*_w_’s for model 16 (calculated by the COSMOtherm program^56^) are included. The COSMO-RS method approaches the experimental value of p*K*_w_ closer than “direct” (not fitted) DFT-based models for all functionals with the exception of M11. The M06-2X, PBE0s, TPSSh, and ωB97X-D functionals reproduce the experimental value also with a very good accuracy of ±0.2 log unit.

**Table 2:** p*K*_w_ at 298 K Obtained within Models 6, 8, 10, and 16 (Values form COSMOtherm)^a^

model	basis set	B3LYP	M11	M11-L	M06-2X	PBE0	revPBE0	TPSSh	ωB97X-D
6	B2	14.7	13.2	17.0	15.4	15.5	15.3	14.6	15.2
8	B3	15.0	13.5	16.8	16.0	15.8	15.6	14.9	15.3
10	B4	16.1	14.7	17.8	17.0	16.9	16.7	15.9	16.4
16		13.3	11.8	15.4	13.8	14.2	14.0	14.2	14.0

a Model 7 provides the same values as model 6 and model 9 and the same data as model 8 since H_3_O^+^ and OH^–^ are the reference ions for s-UAKS cavities. The experimental value of p*K*_w_ is 14.0 at 298.15 K (temperature used for evaluation of the RR-HO model of statistical physics).

### Dissociation Constants of Amino Acids Determined with DFT Functionals

For the lowest lying conformers of each of the amino acids, an SP evaluation of all the solvation models was conducted obtaining the p*K*_a_'s of all the considered groups (carboxyl, amino, and side chains). The corresponding root-mean-square deviations (RMSD) from experimental p*K*_a_ data^1^ are collected in the Table 3. From this table, it can be noticed that the construction of the solvation model has a much larger impact on the accuracy of p*K*_a_ determination than the choice of individual functionals. Regarding individual models, it can be generalized that most of the functionals behave similarly, giving relatively close RMSD values. The only exception from the explored functionals represents ωB97X-D, which does not obey the general outline and leads to visibly higher differences for all the solvation models except model 2. Therefore, its results will be discussed separately. To appraise quality of the individual functionals for p*K*_a_ determination, the weighted average of RMSDs
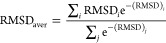
9was calculated for models where the values do not exceed 6 log units. The weights are taken as e^–RMSD^ in order to give a bigger impact of more accurate models on the result. The best performance is exhibited by the M11-L and M06-2X functionals (cf. Table 3). From the “opposite insight” considering sums of the RMSDs over all the functionals, the best solvation model is model 8. The successful models reproducing the experimental p*K*_a_ values of all amino acids with RMSD below 2.5 log units are collected in Table 3. As mentioned already above, the strategy for construction of the implicit solvation model is one of the main factors for the quality of the results. The best results are obtained with models based on the D-PCM, which provide acceptable agreement with experiments.^57^ Using the other implicit solvation models (IEF-PCM, SMD, and C-PCM) leads to an increase of RMSD in the majority of the cases. A compensation of escaped charge from the cavity also plays an important role, especially for anionic and zwitterionic structures.^10,42,58^ Unless compensation of escaped charge is included, the deviations visibly increase except the long-range corrected ωB97X-D functional, as it follows from comparison of models 10 and 11 where the RMSD increases by about 1.0 log unit in average (cf. Table 3). It is worth mentioning that their use is different in the default D-PCM and IEF-PCM settings in Gaussian where the charge compensation is employed using keyword in the D-PCM and in the IEF-PCM model is implemented directly.^63^

**Table 3:** Performance of Models with RMSD below 6 for All the Considered Functionals^a^

model	B3LYP	M11	M11-L	M06-2X	PBE0	revPBE0	TPSSh	ωB97X-D	sum
2	3.36	2.60	2.34	2.37	3.29	3.26	3.41	3.31	23.94
4	3.84	3.09	2.67	2.83	3.77	3.75	3.88	3.83	27.67
6	2.15	2.22	2.21	2.18	2.12	2.11	2.17	4.12	19.27
7	2.28	2.16	1.97	1.79	2.34	2.30	2.46	4.05	19.36
8	2.13	2.08	2.03	2.11	2.07	2.07	2.11	4.01	18.60
9	2.23	2.04	1.90	1.74	2.26	2.23	2.38	3.88	18.66
10	2.51	2.16	1.89	2.18	2.43	2.77	2.51	5.03	21.48
11	3.82	2.96	2.51	2.86	3.71	4.02	3.80	3.76	27.43
12	2.66	2.33	1.90	1.93	2.66	2.93	2.79	4.26	21.45
weighted average^b^	2.45	2.29	2.09	2.09	2.43	2.46	2.51	3.87	
15	15.49	14.74	13.41	14.48	15.15	15.14	15.32	15.41	121.4
16	1.60	1.12	2.47	1.63	2.03	1.94	2.25	2.36	15.40

a Model 15 is added due to relation to model 16.

b Weighted average calculated from models 2, 4, 6, 7, 8, 9, 10, 11, and 12 according to eq 9.

The type of cavity is another factor that substantially influences the quality of results. Rescaling of UAKS atomic radii according to Zimmermann^12,14,15,49^ reduces RMSD of the corresponding models as follows from comparison with nonrescaled models (comparing models 6 vs 8, 10 vs 12, or 8 vs 9). A similar idea of rescaling cavities based on partial charge comes also from Ginovska et al.^59^ where Merz–Singh–Kollman MEP charges^60^ were used for analogous scaling of the UAKS cavities. Cavity rescaling also improves the performance of IEF-PCM solvation models up to 0.5 logarithmic unit (B3LYP and revPBE0). However, functionals of Truhlar’s group (M06-2X, M11, and M11-L) do not benefit from cavity rescaling with either B2 or B3 basis sets. Models with the electrostatic scaling factor α = 1.1 give considerably smaller p*K*_a_ values (models 3 and 5) than pertinent models with the scaling factor α = 1.2 (models 2 and 4); models 3, 4, and 5 are not shown in Table 3 due to substantially higher RMSD. Interestingly, model 5 becomes very accurate after fitting proton solvation energy, which removes the systematic error (see section Fitting of the Proton Solvation Energy).

Considering the effect of basis sets, the largest set (B4) usually deteriorates the accuracy by up to 20–30%. Nevertheless, changes from B2 to B3 sets decrease the RMSD by at most 10%. Similarly to the situation with p*K*_w_’s, a larger basis set shifts p*K*_a_ to higher values in average. In this way, the shift within the B3 set leads to the best p*K*_a_ estimations.

To summarize the effects of individual parameters within the models, the best results are obtained with model 8 for most of the functionals. As mentioned earlier, the ωB97X-D functional does not follow the behavior of the other selected functionals with dependence on the computation setting. It gives the best agreement with experiment within model 2. However, even in this case, the RMSD is still quite high (3.5 log units).

As already mentioned, model 8 gives the generally closest agreement with the experimental data. Several functionals like TPSSh, revPBE0, B3LYP, M06-2X, and M11 are performing practically equally well. Table 3 shows that all functionals (except for ωB97X-D) reproduce p*K*_a_ values with RMSD scoring between 2.0 and 2.2. These results confirm the already mentioned finding that the choice of the functional has a smaller effect on determination of amino acids’ p*K*_a_ and demonstrate the importance of appropriate parameters in the solvation model. In this sense, good results for all functionals are also obtained for other models with the D-PCM version of the solvation model, which gives so far the best results when a compensation of the escaped charge from the cavity is used (ICOMP = 4 in the Gaussian keywords).

As to the other corrections, inclusion of Grimme’s empirical dispersion causes only small changes in the case of calculations with the B3LYP functionals. The difference in RMSD between the models with and without dispersion corrections is below 0.1. For model 8 with the best performance, larger changes in p*K*_a_’s are found for His, Phe, and Lys amino acids where the biggest p*K*_a_ difference is 1.3. Similarly, Wertz’s corrections only slightly shift p*K*_a_ values by 0.14–0.17 log unit. They play a significant role, usually for reactions where the number of molecules changes, but this is not our case.

The most comprehensive insight into the individual p*K*_a_ values is displayed in Table 4 where results for all amino acids obtained using all functionals within model 8 are collected. Similarly, the analogous p*K*_a_’s of all the amino acids for models 2, 9, 10, and 16 are summarized in Tables S1–S4 in the Supporting Information. The individual numbers demonstrate that reproduction of the experimental data is a fairly difficult task. The absolutely smallest RMSD of 1.74 is provided by model 9 in combination with the M06-2X functional, as can be noticed in Table 5. Despite the more important impact of the solvation models, the performance of individual functionals also differs among various amino acids, and results of the best performing functionals deviate sometimes by even more than 1.0 log unit. From a deeper insight into the accuracy of dissociation constants, it can be revealed that p*K*_a_ estimations are closer to the experimental data for deprotonation of 1-carboxyl groups and/or α-amino groups than for side chains. The most probable reason dwells in a much larger flexibility of side chains and thus higher uncertainty in determination of the most frequent side-chain conformations that influence the final value of p*K*_a_.^29^ From this perspective and considering RMSD as a scoring function, the accuracy of the prediction of the 1-carboxyl group p*K*_a_’s is about 1.0 for the most successful model (8) and the best of the functionals (M06-2X and M11; M11-L deviates slightly more, ca. 1.5), cf. Table 5. The RMSD scoring is nearly twice higher for p*K*_a_ of the α-amino groups, where the corresponding values vary around 2.0 using the same model. However, the scoring is on average about 3.8 log units for p*K*_a_‘s of side chains. In analogy with 1-carboxyl groups, the p*K*_a_ of side-chain carboxyl groups (Asp and Glu) are determined with a somewhat better accuracy (ca. 2.3 log units) than p*K*_a_‘s of remaining side chains where dissociation takes place on side chains with imino or amino groups (His, Arg, and Lys, cf. Table 4 and in details in Table S5). These constants exhibit an RMSD of ca. 4.2 log units. In this case, the magnitude of deviation can be influenced by experimental reference, too. For the Arg guanidine group, the CRC tables report the value of 12.1 log units, which is quite low in comparison with results appearing in other experimental studies: 12.48,^53^ 13.2,^54^ or 13.8.^55^ The latter results are closer to our calculations where the corresponding averaged p*K*_a_'s are 19.8 and 15.9 for models 8 and 9, respectively. From this perspective, some thermodynamical averaging over a higher number of structures based on, e.g., classical MD simulations with a reliable force field, or some short Born–Oppenheimer MD using the DFT method with some fast functional would bring calculated p*K*_a_ to better accord with measured values.

**Table 4:** Comparison of p*K*_w_ and p*K*_a_ Values for All Amino Acids Obtained with All Functionals and the Best Model 8^a^

	**Ala**		**Arg**			**Asn**		**Asp**			**Cys**			**Glu**		
***Exp.***	**2.3**	**9.7**	**2.0**	**9.0**	**12.1**	**2.2**	**8.7**	**2.0**	**9.7**	**3.7**	**1.9**	**10.3**	**8.1**	**2.2**	**9.6**	**4.2**
B3LYP	3.3	9.3	0.5	6.8	20.6	2.8	11.2	3.4	14.1	4.7	1.3	13.6	9.5	2.9	10.2	3.9
B3LYP-D	3.6	9.0	0.5	6.8	20.8	2.8	11.0	3.2	14.1	4.8	1.3	13.6	9.5	2.9	9.9	4
M11	2.3	8.0	–1.8	6.9	18.6	1.5	10.1	1.8	12.1	3.2	0.8	12.7	7.3	0.7	9.2	2.9
M11-L	2.6	6.6	0.3	5.9	15.0	2.4	9.0	2.3	11.4	4.8	2.0	10.5	4.6	1.0	8.1	4.4
M06-2X	3.0	7.3	–0.7	5.4	19.1	2.1	9.3	2.0	11.4	4.8	1.0	11.7	7.4	2.1	8.6	2.9
PBE0	2.8	9.5	0.1	6.7	20.4	3.1	11.2	3.1	13.8	4.3	1.5	13.8	8.0	2.3	9.9	4.6
revPBE0	2.9	9.3	0.2	6.6	20.6	3.1	11.2	3.0	13.8	4.3	1.4	13.7	8.1	2.2	9.7	4.7
TPSSh	2.9	9.6	–0.3	7.2	20.4	2.8	11.4	3.0	14.3	4.2	1.3	14.2	8.6	2.3	10.8	3.7
ωB97X-D	3.7	8.9	0.5	6.9	21.1	2.5	11.0	3.6	12.2	4.4	2.2	13.3	4.4	2.3	9.9	3.9

	**Gln**		**Gly**		**His**			**Ile**		**Leu**		**Lys**			**Met**	
***Exp.***	**2.2**	**9.0**	**2.3**	**9.6**	**1.7**	**9.1**	**6.0**	**2.3**	**9.6**	**2.3**	**9.6**	**2.2**	**9.2**	**10.7**	**2.2**	**9.1**
B3LYP	2.6	7.8	2.9	8.9	1.0	8.4	8.5	1.9	9.0	0.7	9.8	1.2	7.2	11.0	1.7	7.0
B3LYP-D	2.9	8.1	2.8	8.9	1.8	8.9	9.1	1.5	9.3	0.7	9.6	0.2	8.5	10.7	1.8	7.2
M11	1.2	7.4	1.4	8.2	–0.3	7.7	7.6	0.4	7.7	–1.1	9.0	0.3	5.6	11.0	–0.3	5.8
M11-L	1.7	7.5	2.2	7.3	1.3	7.4	3.6	0.0	7.2	–0.7	7.6	0.9	5.6	7.1	0.7	6.2
M06-2X	2.0	6.4	2.6	7.7	0.5	6.9	5.9	1.8	6.8	0.4	7.6	–0.6	5.6	9.6	1.1	5.0
PBE0	2.0	9.0	2.7	8.9	1.7	8.6	7.2	1.8	9.0	0.0	9.6	0.8	7.7	10.4	0.9	7.1
revPBE0	2.0	9.0	2.6	9.0	1.6	8.5	7.2	1.8	9.0	0.0	9.6	0.8	7.7	10.3	1.7	7.0
TPSSh	2.4	9.0	2.4	9.6	1.3	9.2	7.3	1.4	9.8	0.2	10.6	0.5	8.0	10.8	1.1	7.6
ωB97X-D	2.3	9.0	2.6	9.2	1.1	8.6	8.4	1.9	8.8	0.9	9.4	1.0	7.8	11.5	1.4	7.5

	**Phe**		**Pro**		**Ser**		**Thr**		**Trp**		**Tyr**			**Val**		**p***K*_**w**_
***Exp.***	**2.2**	**9.1**	**2.0**	**10.5**	**2.1**	**9.1**	**2.2**	**9.0**	**2.4**	**9.3**	**2.2**	**9.0**	**10.1**	**2.3**	**9.5**	**14.0**
B3LYP	2.5	9.9	0.6	13.1	3.2	11.4	2.8	10.9	2.7	9.5	2.6	10.4	16.6	2.4	9.7	15.0
B3LYP-D	2.5	8.9	0.4	13.1	3.2	11.3	2.6	10.7	2.3	9.6	2.7	10.0	16.4	2.3	9.8	15.0
M11	1.0	8.7	–1.2	12.9	1.6	10.7	1.3	11.1	0.5	9.8	1.4	9.2	14.9	0.8	8.7	13.5
M11-L	0.4	8.0	0.2	8.5	2.7	9.2	1.4	8.7	0.9	7.7	0.9	7.4	14.8	1.1	7.1	16.8
M06-2X	1.8	7.2	–0.2	10.9	2.5	9.7	1.8	10.7	1.6	8.6	1.4	8.7	15.9	1.8	7.7	16.0
PBE0	2.1	9.5	0.4	12.5	3.1	11.3	2.4	11.5	2.3	10.0	1.8	10.2	16.0	1.8	9.8	15.8
revPBE0	2.0	9.4	0.3	12.6	3.1	11.2	2.3	11.3	2.3	10.1	1.7	10.2	16.0	1.9	9.7	15.6
TPSSh	2.1	9.2	0.4	13.1	2.9	11.8	1.9	12.0	2.7	10.4	1.3	10.7	15.9	1.8	10.2	14.9
ωB97X-D	2.2	18.6	0.4	12.9	3.0	11.3	2.2	11.3	3.2	9.8	1.5	10.5	16.6	2.4	9.3	15.3

a The third column (if present) correpsponds to side-chain p*K*_a_ values.

**Table 5:** Individual RMSDs of p*K*_a_ Values for 1-Carboxylic Groups, α-Amino Groups, and Side Chains of All Relevant Amino Acids^a^

	model 8				model 9				COSMO-RS			
functional	COOH	NH_2_	side	total	COOH	NH_2_	side	total	COOH	NH_2_	side	total
B3LYP	0.87	1.90	4.29	2.13	0.95	2.80	2.99	2.23	2.22	0.61	1.52	1.60
M11	1.84	1.85	3.19	2.02	1.89	2.18	2.12	2.04	1.14	0.90	1.36	1.12
M11-L	1.33	2.03	3.21	2.03	1.46	1.36	3.52	1.90	3.64	0.73	1.46	2.47
M06-2X	1.21	2.15	3.57	2.11	1.24	1.67	2.79	1.74	2.14	1.11	1.25	1.63
PBE0	0.98	1.82	4.09	2.07	1.26	2.73	2.96	2.30	2.80	0.93	1.82	2.03
revPBE0	0.94	1.81	4.13	2.07	1.23	2.68	2.98	2.20	2.68	0.88	1.77	1.94
TPSSh	1.03	2.03	3.95	2.11	1.35	3.03	2.68	2.40	2.88	1.55	2.03	2.25
ωB97X-D	0.89	6.79	4.63	4.77	1.05	5.57	3.40	3.90	3.19	1.22	2.19	2.36
average	1.14	2.55	3.88		1.30	2.75	2.93		2.59	0.99	1.68	

a The s-UAKS model is used in model 8, while the original UAKS model is employed in model 9. The COSMOtherm calculations (model 16) are based on the COSMO model 15.

The p*K*_a_ estimation of the side chain of Tyr is another problematic case. Generally, the results of our computational models are visibly higher than the experimental value of 10.1. It indicates that the deprotonated form should be a little bit more energetically stable. This means that its total electronic energy should be lower, which can be reached by a larger reduction of the sphere in the Tyr oxygen anion within the cavity construction. The smaller radius on the oxygen atom induces a larger surface charge on the cavity and thus increased electrostatic interaction between the cavity and negatively charged oxygen. In this way, the solvation energy would lead to a more negative electronic energy (higher stabilization). Such considerations are supported by the fact that when the standard UAKS model is used, a formal (more) negative charge (−1.00) is applied, and thus, the shorter oxygen radius leads to larger cavity reduction resulting in even larger lowering of the electronic energy of this form. Therefore, a closer p*K*_a_ value to experiment is obtained in this case. It looks like the natural population analysis (NPA in the NBO procedure) underestimates the negative charge of the corresponding oxygen atom in the side chain of Tyr. For the best model (8), a value of 14.8 was obtained using the functional M11-L. Using the B3LYP functional and model 5 (based on formal charges) gives p*K*_a_ = 7.1, i.e., 3 log units lower than the experiment. However, this model gives lower p*K*_a_ values systematically, cf. further discussion below.

Cys and His are the only amino acids whose side chain deprotonates in the range of physiological pH. Hence, a correct reproduction of these p*K*_a_’s is particularly important for biological studies. Their calculated p*K*_a_’s are collected in Table 6 for the best performing model 8 and all the explored functionals. In the case of Cys, calculations using PBE0, revPBE0, and partially also TPSSh functionals are in very good accord with the experimental value of 8.14. These three functionals also match fairly well in estimation of the p*K*_a_ value of the imidazole ring of His with the experimental p*K*_a_ = 6.0. Focusing on the p*K*_a_ of this group, another good option represents the M06-2X functional (p*K*_a_ = 5.9), which exhibits also the lowest RMSD scoring in comparison with all other functionals in Table 3.

**Table 6:** Comparison of p*K*_a_ for Cys and His Obtained within the Best Model 8 for All Explored Functionals

	Cys			His			
	COOH	SH	NH_2_	COOH	imidazole	NH_2_	RMSD
exp.	1.9	8.1	10.3	1.7	6.0	9.1	
B3LYP	1.3	9.5	13.6	1.0	8.5	8.4	1.85
M11	0.8	7.3	12.7	–0.3	7.6	7.7	1.64
M11-L	2.0	4.6	10.5	1.3	3.6	7.4	1.88
M06-2X	1.0	7.4	11.7	0.5	5.9	6.9	1.26
PBE0	1.5	8.0	13.8	1.7	7.2	8.6	1.53
revPBE0	1.4	8.1	13.7	1.6	7.2	8.5	1.51
TPSSh	1.3	8.6	14.2	1.3	7.3	9.2	1.72
ωB97X-D	2.2	4.4	13.3	1.1	8.4	8.6	2.20

**Figure 1:**
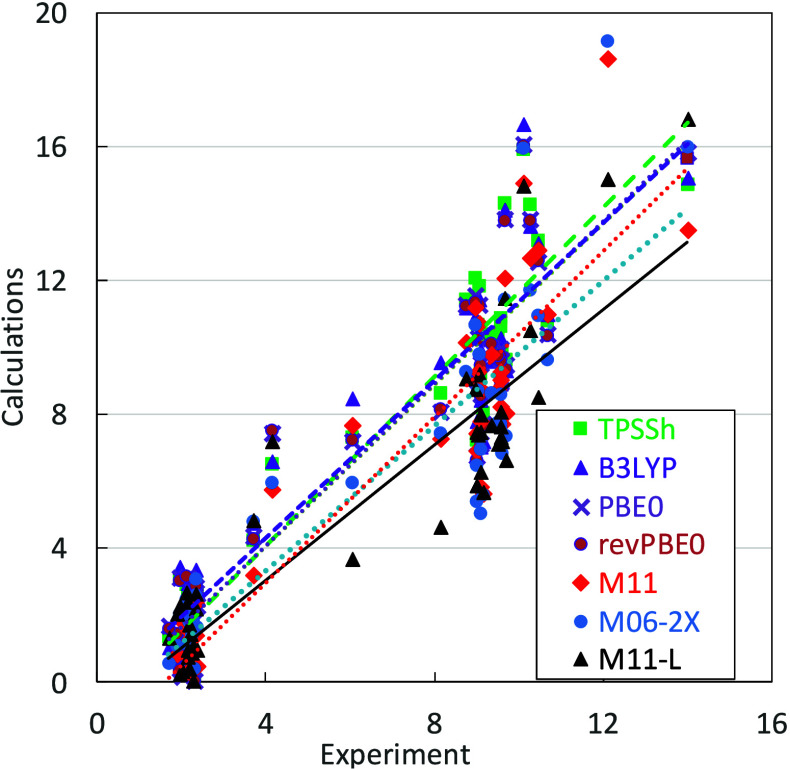
**:** Comparison of experimental and computed values of p*K*_a_ for all amino acids and water for all the functionals (except ωB97X-D) and model 8.

In order to demonstrate the behavior of model 8, we present a plot of computed p*K*_a_ values vs experimental data employing all the considered functionals with the exception of ωB97X-D (Figure 1). p*K*_a_ points for individual functionals were fitted by lines, and parameters of the linear fit are summarized in Table 7. In the optimal case, they should correspond to the line *y* = *x*. As can be seen from Table 7, the best functional M11-L really has its slope (1.0082) very close to 1.0; nevertheless, its intercept is also close to 1.0. The last set of points in Figure 1 (with values close to 14) corresponds to the ionic product of water, where all of the functionals managed to fit the experimental value reasonably well. On the contrary, only the three “lowest” points (M11-L, M11, and M06-2X) can be found in the displayed range for the Arg side chain (guanidine group close to a value of 12). Here, the error in p*K*_a_ determination is one of the largest, see also Table 4. In the middle of Figure 1, the other four series can be easily recognized, namely, side-chain carboxyls of Asp and Glu (3.7 and 4.2, respectively), the amino group of His (6.0), and the thiol group of Cys (8.1). For the latter two, Figure 1 clearly documents larger scattering of the obtained values in comparison with more compact values for p*K*_a_ determined for 1-carboxylic groups, as discussed above.

**Table 7:** Parameters of Linear Dependency between Calculated (*y*) and Experimental (*x*) p*K*_a_ (*y* = *ax* + *b*)

	*a*	*b*
PBE0	1.2128	–0.7757
M11	1.2377	–1.9717
M11-L	1.0082	–0.9794
B3LYP	1.1728	–0.3629
M06-2X	1.0814	–0.9968
revPBE0	1.2058	–0.7497
TPSSh	1.2651	–0.9808

### Fitting of the Proton Solvation Energy

In addition to the direct calculations, the p*K*_a_ values were also obtained by fitting the parameter *b* in eq 8 (keeping parameter *a* constant in the first step) for each considered model based on the calculated differences of chemical potentials Δ*G*^0^ of the pertinent protonated and deprotonated forms. The fitted parameter *b* involves solvation Gibbs free energy of the proton (Δ*G*_solv_(H^+^)), the contribution to the Gibbs free energy from translational motion of the proton, energy related to the change of concentration (*RT* ln(*V*_M_)), and the term −log[H_2_O]/(2.303*RT*) as mentioned in computational details. In addition to these contributions with physical meaning, they also contain a systematic error of the individual models, which is subsequently transferred to the calculated proton free solvation energy. Nevertheless, the extracted proton solvation free energies Δ*G*_solv_(H^+^) from parameter *b* (up to a systematic deviation) can be found in the range from −269.9 to −262.1 kcal·mol^–1^ for the best models (cf. Table 8), which closely approaches the experimental value of −265.9 kcal·mol^–1^.

**Table 8:** RMSD for Model 5 from the Fitted Procedure; Parameter *b* and Calculated Gibbs Free Energy of Proton Solvation^a^

	fitting					
functional	model 5	*b*^a^	Δ*G*_sol_ (H^+^)^a^	best model^b^	direct	COSMO-RS
B3LYP	1.77	–274.0	–267.2	1.74 (3)	2.13 (8)	1.60
M11	1.66	–273.0	–266.2		2.04 (9)	1.72
M11-L	2.32	–272.8	–266.0	1.70 (15)	1.89 (10)	2.47
M06-2X	1.92	–268.9	–262.1	1.42 (15)	1.74 (9)	1.63
PBE0	1.73	–275.7	–268.9		2.07 (8)	2.03
revPBE0	1.73	–275.5	–268.8		2.07 (8)	1.94
TPSSh	1.62	–276.4	–269.7		2.11 (8)	2.25
ωB97X-D	1.60	–276.7	–269.9		3.31 (2)	2.36

a Fitted parameters for the best model are in kcal·mol^–1^.

b RMSD in the case when the best model does not correspond to model 5; the number of the best model is in parentheses. The RMSDs for the best “direct” and COSMO-RS calculations are appended for comparison.

From the viewpoint of p*K*_a_ values, the fitting provides visible improvement (cf. Table S7). For all the regarded functionals, lowering of the RMSD scoring below 1.75 is demonstrated in Table 8 and Table S8 along with the pertinent parameter *b*. The M06-2X functional in combination with C-PCM/Klamt (model 15) leads to an even lower RMSD value of 1.42. This represents a reduction of RMSD by 18% between the best methods in “direct” and fitted schemes (p*K*_a_ calculations using eqs 3 and 8). The most accurate results based on the fitting of the parameter *b* are reached within model 5 where the IEF-PCM/UAKS model is utilized, with the exception of functionals M11-L and M06-2X (cf. Table S7). This is interesting since in the direct calculations, model 5 with the scaling constant α = 1.1 strongly and systematically underestimates the p*K*_a_ values, cf. Table S6 where mean signed errors (MSEs) are always close to −8 (and the same is true also for model 3). On the other hand, the improvement obtained by fitting is only marginal in the case of the most accurate models from “direct” calculations (like model 8) since their MSEs are small and the fitting of the constant term in eq 8 does not lead to any significant improvement (cf. Table 9 and Table S6).

**Table 9:** RMSDs for Model 8 from the “Direct” Calculations and from the Fitting together with MSE from the “Direct” Calculations, the Fitted Parameter *b*, and Calculated Proton Solvation Energy in kcal·mol^–1^

functional	RMSD (direct)	MSE (direct)	RMSD (fitting)	*b*	Δ*G*_sol_ (H^+^)
B3LYP	2.13	0.7	2.02	–267.5	–260.7
M11	2.08	–0.5	2.04	–265.9	–259.2
M11-L	2.03	–0.9	1.73	–268.5	–261.7
M06-2X	2.11	–0.5	2.04	–266.2	–259.5
PBE0	2.07	0.6	2.01	–268.8	–262.0
revPBE0	2.07	0.5	2.01	–268.6	–261.8
TPSSh	2.11	0.7	2.02	–269.8	–263.0
ωB97X-D	4.77	1.2	4.71	–270.7	–263.9

Fitting of both linear parameters (*a* as well as *b*) in eq 8 further reduces RMSDs for all models (cf. Table S9), but the fitted parameters substantially deviate from the physical values in this case (cf. Table S10). For example, the lowest obtained RMSD of 0.63 is determined by fitting within model 15 (C-PCM/Klamt) and using the TPSSh functional leading to parameters *a* = 0.463 kcal^–1^·mol and *b* = −123.9 kcal·mol^–1^. This means that the final parameters are roughly two-thirds and a half of their physical values, respectively. If one would use this in calculations, model 15 represents the best option providing RMSDs of around 0.7 for most of the considered functionals, B3LYP, M11, PBE0, revPBE0, TPSSh, and ωB97X-D (cf. Table S10). For comparison, the calculated data are collected in Table S11.

### COSMOtherm Results

Using the COSMOtherm program, the p*K*_a_ values are determined based on the difference of Gibbs free energies of protonated (AH) and deprotonated (A^–^) amino acid forms according to the following equation:
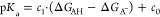
10where *c*_0_ and *c*_1_ are fitted coefficients for different solvents and conditions. The total Gibbs free energies are calculated within the COSMO-RS preparation file within evaluation of model 15, using their energies and conductor polarization charge densities. Such a p*K*_a_ calculation is close to the model from the previous part where both parameters *a* and *b* are fitted. Nevertheless, it does not significantly improve results for all functionals. If the RMSDs from COSMOtherm calculations are compared with the best model in the “direct” calculations (using eq 3), similar deviations are obtained for the M06-2X, PBE0, revPBE0, and TPSSh functionals, and even a substantially higher RMSD can be noticed for the M11-L functional (cf. Table 8). The recommended functional, which should be used with COSMOtherm according to its manual,^46^ is BP86 in the Turbomole program and should lead to highly accurate data. The p*K*_a_ values determined by fitting the *b* parameter in eq 8 are comparable to the COSMOtherm calculations.

Focusing on the origin of deviations of the COSMOtherm values in detail, a decrease of the RMSD is especially observed for p*K*_a_ values of α-amino and side-chain groups, which are reduced by 1.55 and 2.21 log units, respectively (cf. Table 5). This is valid for most of the regarded functionals relative to the best “direct” model 8. On the other hand, the accuracy of acid dissociation constants of 1-carboxyl groups from COSMOtherm is lower than for the PCM models with the UAKS and s-UAKS cavities exhibiting an average RMSD increase of 1.45 log units, except the M11 functional (cf. Table 5 or Table S6). Generally, the accuracy of p*K*_a_ evaluation of the 1-carboxylic groups is decisive for the resulting magnitude of RMSD in the COSMOtherm calculations, and it simultaneously represents an advantage of PCM models with scaled radii, especially with the s-UAKS cavities.

### Predictions of p*K*_a_ at the Post-HF Levels: The CCSD(T) Calculations

In order to relate our DFT calculations to a standard theoretical reference, except the experimental data, the CCSD(T) calculations were also performed with three different settings, CC_1, CC_2, and CC_3 collected in Table 1. As one could expect, addition of correlation energy to HF approximation generally improves performance of all the post-HF levels available from CCSD(T) calculations (MP2, MP3, MP4SDQ, CCSD, and CCSD(T)). Comparison of RMSDs for the individual ab initio levels is summarized in Table 10. It shows that RMSDs for solvation models CC_1 and CC_2 exhibit a lower value at the MP2 level of correlation energy than at CCSD(T), and for the CC_3 setting, both correlation levels are practically the same. Nevertheless, the higher orders of perturbation theory lead to an additional increase of the RMSDs again.

**Table 10:** Total RMSDs and MSEs from CCSD(T) Calculations at Different Post-HF Levels with Different Solvation Models

	RMSD				MSE		
	CC_1	CC_2	CC_3		CC_1	CC_2	CC_3
HF	3.74	2.95	2.80		2.81	1.14	0.91
MP2	2.75	1.99	2.09		1.84	–0.12	–0.28
MP3	3.71	2.55	2.30		2.81	0.92	0.81
MP4SDQ	3.42	2.30	2.14		2.63	0.75	0.57
CCSD	3.54	2.43	2.21		2.72	0.85	0.69
CCSD(T)	3.21	2.16	2.06		2.36	0.44	0.25

The lowest RMSD of 2.06 was reached at the CCSD(T) level with CC_3 settings and is comparable with most of the DFT functionals, cf. Table 3. Also, the influence of the considered solvation models and basis sets follows the analogous trends, as discussed above for the DFT calculations. The D-PCM solvation model used within CC_2 and CC_3 models clearly gives a more accurate description of p*K*_a_ values at all available post-HF levels in comparison with the IEF-PCM model (cf. Table 10). The differences in the RMSDs at the corresponding levels are higher than 0.75 log unit. The impact of the size of the basis set on accuracy of results is similar to the basis set influence on the ionic product of water where the MSE increases with larger basis comparing CC_2 and CC_3, cf. Table 10. The differences of calculations with B2 and B3 basis sets are small nevertheless; the B2 basis set gives slightly better results. Thus, it can be repeated that the choice of the solvation model plays the crucial role for accuracy of the p*K*_a_ calculations.

## Conclusions

In this study, the strong dependence of computed p*K*_a_ values on computational settings is demonstrated for proteinogenic amino acids. The choice of the implicit solvent is the key factor influencing results of the calculations. The D-PCM model provides superior agreement with experimental data in comparison with the other solvation models. Moreover, according to our experience and accented by these results, the strategy for cavity construction is also a very important factor. Models with the UAKS cavity and the electrostatic scaling factor of 1.2 give higher values of p*K*_a_ and usually better agreement with experiment than analogous models with a scaling factor of 1.1. Nevertheless, using a scaling factor of 1.2 and rescaling atomic radii according to partial charges of atoms like oxygen, sulfur, chlorine, and others make p*K*_a_ predictions even closer to experimental data. Our rescaling technique is based on the original idea of Orozco et al.^61^ and Tomasi et al.^22^ Therefore, we did not rescale nitrogen atoms, which would obviously lead to better estimation of the p*K*_a_ values for side-chain nitrogen groups like in His, Arg, or Lys. The choice of the DFT functional affects the p*K*_a_ value to a surprisingly smaller extent. In fact, RMSD scoring of the best model (model 8) obtained by B3LYP, M11, M11-L, M06-2X, PBE0, revPBE0, and TPSSh functionals differs only by up to 0.1 log unit as can be seen in Table 3. Concerning the models with DFT functionals, the best results are obtained at the M06-2*X*/6-311++G**/D-PCM/UAKS level^12,14,15,49^ (the M06-2X functional in combination with model 9), which leads to RMSD = 1.74 log units. Comparable RMSD scoring was also obtained for CCSD(T) and MP2 correlation levels of ab initio calculations using the solvation model (8) labeled as CC_3. The “direct” DFT results are of comparable quality to the COSMO-RS model as implemented in the COSMOtherm program. However, when a similar procedure (like in COSMOtherm) is applied for fitting parameters from eq 8, even more accurate results are obtained for practically all of the functionals.

As discussed above, evaluation of p*K*_a_’s of 1-carboxyl groups is more accurate than results of α-amino groups. Estimation of the p*K*_a_’s of the amino acid side chains exhibits the highest RMSD scoring. Regarding p*K*_a_ of Cys and His side chains, the only two amino acids dissociating at physiological pH, all functionals provide fair agreement with the experimental data with RMSD up to 1.5 log unit.

## Supporting Information Available

Short introduction to PCM methods and compensation of escaped charge from the cavity, complete p*K*_a_ values evaluated for the best models in the “direct” calculations and using the COSMOtherm program (models 2, 9, 10, and 16), and some additional tables with RMSD and MSE values of individual groups (carboxyl, amino, and side chains) as mentioned in the text (PDF)
