# Supplementary material for: Determination of Amino Acids’ pKa: Importance of Cavity Scaling within Implicit Solvation Models and Choice of DFT Functionals
Source: J Phys Chem B. 2024 Feb 12;128(7):1627–37. doi: 10.1021/acs.jpcb.3c07007 (PMC10895671; doi:10.1021/acs.jpcb.3c07007)
Supplement: Supplementary file 1 — jp3c07007_si_001.pdf [file jp3c07007_si_001.pdf]

Determination of Amino Acids pK_a: Importance of Cavity Scaling within Implicit Solvation Models and Choice of DFT Functionals.

*Filip Šebesta, Žofie Sovová, Jaroslav V. Burda**

[1]Department of Chemical Physics and Optics, Faculty of Mathematics and Physics, Charles University, Ke Karlovu 3, 121 16 Prague 2, Czech Republic.

*corresponding author: burda@karlov.mff.cuni.cz

Supplementary materials

1. Short introduction to PCM methods and compensation of escaped charge from the cavity

2. Calculated pK_a values for the best models in the 'direct' calculations, using the COSMOtherm program

A detailed summary of calculated pK_a values for the best solvent models:

a) model 9 - **Table S1**,
b) model 10 - **Table S2**,
c) model 2 - **Table S3**, and
d) model 16: COSMO-RS calculations in the COSMOtherm program - **Table S4**

3. MSEs and RMSDs for selected models and specific pK_a values

Mean signed errors (MSEs) for all considered models and root mean square deviations (RMSDs) for pK_a values belonging to specific groups (carboxyl, amino, and side chain groups) collected in **Tables S5** and **S6**. The deviations for fitted models are also presented along with fitted parameters for the best models and determined pK_a values (**Tables S7-S11**). A description of the employed models can be found in the main paper in section *Computational details*.

Table S5: Total RMSDs and RMSDs for specific pK_a values within selected solvent models. 'COOH' means all 1-carboxyl groups, 'NH_2' α-amino groups and 'side-chain' acidic or basic groups of side-chains of Arg, Asp, Cys, Glu, His, Lys, Tyr. For side-chain pK_a values another splitting is consider: 'side COOH' – carboxyl groups of Asp and Glu, 'side NH_2' – amino groups of Arg, His, and Lys, 'side SH' - thiol group of Cys, and 'side OH' – hydroxyl group of Tyr.

Table S6: MSEs of pK_a values calculated using all regarded functionals and solvent models.

Table S7: RMSDs of pK_a values calculated using **Equation 8** when only parameter *b* is fitted. Data are presented for all regarded functionals and selected models. Lowest RMSDs for the individual functionals are in bold.

Table S8: pK_a values for all amino acids obtained by fitting parameter *b* in **Equation 8** and using difference in Gibbs free energy ΔG^0 between the protonated and deprotonated forms calculated within model *5* using all considered functionals. Parameter *a* is 0.73305 mol·kcal^{-1} and fitted parameter *b* in kcal·mol^{-1} is stated in the last column.

Table S9: RMSDs of pK_a values calculated using **Equation 8** when both parameters *a* and *b* are fitted. Data are collected for all regarded functionals and selected models.

Table S10: Fitted parameters *a* and *b* in **Equation 8** within model *15* for the individual functionals. They were used for determination of pK_a values in **Table S11**.

Table S11: pK_a values for all amino acids obtained by fitting both parameters *a* and *b* in **Equation 8** and using the difference in Gibbs free energy ΔG^0 between the protonated and deprotonated forms calculated within model *15* for all considered functionals. The calculated RMSDs for the given functionals are stated in the last column.

1. Used PCM models and their differences

In this section, we provide a brief overview of used PCM methods while a detailed description can be found elsewhere.[1-4] We mostly take over from ref. [1]

In order to involve interaction with surrounding solvent, a solute is embedded into a cavity in dielectric continuum in PCM methods. In this case, an electrostatic solute-solvent interaction is given by potential V_σ from induced charges $\sigma(\vec{s})$ on the cavity surface and solute charge distribution $\rho(\vec{r})$

$$E_{solute-solvent} = \frac{1}{2}\int \rho(\vec{r})V_\sigma(\vec{r})d\vec{r}$$

which leads to polarization of solute electronic wavefunction. For used methods and non-ionic solvents with permittivity ε, the surface charge $\sigma(\vec{s})$ is obtained as solution of Poisson's equation:

$$-\nabla(\varepsilon(\vec{r})\nabla V(\vec{r})) = 4\pi\rho(\vec{r})$$

with boundary conditions on the cavity surface Γ

$$V_{in}(\vec{s}) = V_{out}(\vec{s}) \qquad (potential\ continuity)$$

$$\frac{1}{\varepsilon}\frac{\partial}{\partial\vec{n}}V_{in}(\vec{s}) = \frac{\partial}{\partial\vec{n}}V_{out}(\vec{s}) \qquad (jump\ condition)$$

The non-electrostatic contribution to solvation energy is normally decomposed to repulsion, dispersion and cavity formation energy that are evaluated based on the works of Floris[5] and Pierotti.[6]

D-PCM

The original formulation, now called D-PCM, can be derived for constant isotropic permittivity ε from electrostatic potential V and polarization vector:

$$\vec{P_a}(\vec{r}) = \frac{\varepsilon_a - 1}{4\pi}\nabla V(\vec{r})\ .$$

The index a corresponds to a given material. From (macroscopic) Gauss's law and the jump condition, the surface charge can subsequently be determined as

$$\sigma_{ij} = -(\vec{P_j} - \vec{P_i})\cdot\vec{n}_{ij} = \frac{\varepsilon - 1}{4\pi\varepsilon}\frac{\partial}{\partial\vec{n}}(V_M + V_\sigma)$$

where $\varepsilon_i = \varepsilon_j = 1$ was used in the definition of polarization vectors $\mathbf{P}_{in}$ and $\mathbf{P}_{out}$. V_M is the potential generated by the solute and V_σ by the surface charges. This equation can be rewritten in the form $\mathbf{q} = -\mathbf{Kf}$ for a set of point charges on cavity surface ($q_k = \sigma(\vec{s})A_k$, A_k is the area of the surface fragment) where

$$\boldsymbol{K} = \left(2\pi\frac{\varepsilon+1}{\epsilon-1}\boldsymbol{A}^{-1} - \boldsymbol{D}^*\right)^{-1} \text{ with } D_{ij} = \frac{(\vec{s}_i - \vec{s}_j)\cdot\vec{n}_j}{|\vec{s}_i - \vec{s}_j|^3}$$

$$\boldsymbol{f} = E_n = -\frac{\partial}{\partial\vec{n}}V_M$$

E_n is the normal component of the electric field and A a matrix containing areas of surface fragments on the diagonal. Such a form is used in QM programs.

IEF-PCM

The integral equation formalism (IEF) works with electrostatic potentials against the original D-PCM method where the normal component of the electric field represents fundamental property. This reformulation is more general and can be used for anisotropic and ionic liquids (when Poisson-Boltzmann equation is considered). Derivation of working equations starts by splitting the total potential into potential V_M generated by charge density of a molecule and potential V_R generated by charges induced in the dielectric – reaction potential. They are defined using the Green kernels and generating charge distributions ρ_x:

$$V_X = \int G(x,y)\rho_x(y)dy$$

An important result is that V_R can be represented as a single layer potential. Using the Poisson's equation, the jump condition, and boundary conditions at infinity $\lim_{r\to\infty} V(\vec{r}) = 0$ again leads to final equations in the form **q** = -**Kf** with

$$\boldsymbol{K} = \left\{\left(2\pi\frac{\varepsilon+1}{\epsilon-1}\boldsymbol{A}^{-1} - \boldsymbol{D}\right)S\right\}^{-1}(2\pi\boldsymbol{A}^{-1} - \boldsymbol{D}) \text{ where } S_{ij} = \frac{1}{|\vec{s}_i - \vec{s}_j|}$$

$$f = V_M$$

This formalism also includes an approximation of dielectric volume polarization.[7]

COSMO and C-PCM

Another approach dwells in setting ε to infinity and in such a way moving from dielectric surroundings into a conductor. Equations become simpler as the total electric field in the conductor as well as the electrostatic potential is zero. This leads to charges on the cavity surface:

$$\boldsymbol{q}^* = -\boldsymbol{S}^{-1}\boldsymbol{V_M}$$

where ***S*** is defined in the previous equation. Nevertheless, we would still like to obtain response from dielectric surroundings so calculated charges q^* are scaled as

$$q_k = f(\varepsilon)q_k^* \quad \text{with} \quad f(\varepsilon) = \frac{\varepsilon-1}{\varepsilon+x}\ .$$

In the COSMO (Conductor-like Screening Model) model, the x is set to 0.5. Another implementation is known as C-PCM where $x = 0$ in the scaling functions.

COSMO-RS

COSMO-RS (COSMO for real solvents) method represents a model that is based on the statistical physics and includes molecular concentration of compounds in solution. It struggles to find the best contact interaction between molecular groups in the sense that molecular surface parts which are in contact should ideally correspond to charge density of the same size but opposite sign. In order to find them, calculated surface charges from the COSMO method are employed and surfaces are divided into small segments with charge density σ. From a histogram corresponding to number of segments with charge σ, so-called σ-profile $p_s(\sigma)$, the chemical potential of a surface segment with charge σ is subsequently determined as

$$\mu_s(\sigma) = -\frac{kT}{a_{eff}}\ln\left\{\int d\sigma' p_s(\sigma')\cdot e^{\frac{-a_{eff}(e_{int}(\sigma,\sigma')+\mu_s(\sigma'))}{kT}}\right\}$$

where $e_{int}(\sigma,\sigma')$ is the surface contact energy between segments with polarization charges σ and σ' and a_{eff} an effective contact area. Having $\mu_s(\sigma)$ in hands, one can calculate the chemical potential of the molecule M in solvent S using

$$\mu_S^M = \int d\sigma\, p^M(\sigma)\, \mu_S(\sigma) + k_b T \ln\{x + \gamma_{comb,S}^M\} \quad .$$

The second term includes information about mole fraction x and a 'combinatorial term' which represents a correction for relative sizes of the solvent and the solute. Finally, we can evaluate solvation Gibbs free energy from the pure compound gas phase to the solvated phase:

$$\Delta G_{gas \rightarrow solv} = \mu_{solv} - \mu_{gas} + RT \ln \frac{V_{solv}}{V_{gas}}$$

and solvation Gibbs free energy from the pure compound liquid phase to the solvated phase

$$\Delta G_{liq \rightarrow solv} = \mu_{solv} - \mu_{pure}$$

where μ_{gas} (μ_{pure}) is the chemical potential of the pure compound in gas (liquid) phase and μ_{solv} is the chemical potential of the compound in a liquid phase.

SMD

SMD[8] model is built on the IEF-PCM approach for electrostatic interaction with dielectric bulk (ΔG_{EP}). Nevertheless, (non-electrostatic) contributions from solvent cavitation, dispersion, and changes in solvent local structure (G_{CDS}) are calculated based on the solvent accessible surface area A_k and atomic and molecular solvent tensions σ_k and $\sigma^{[M]}$. The latter is determined as

$$\sigma_k = \tilde{\sigma}_{Z_k} + \sum_{k'}^{atoms} \tilde{\sigma}_{Z_k Z_{k'}} T_k \ ,$$

$$\sigma^{[M]} = \tilde{\sigma}^{[\gamma]} \frac{\gamma}{\gamma_0} + \tilde{\sigma}^{[\varphi^2]} \varphi^2 + \tilde{\sigma}^{[\psi^2]} \psi^2 + \tilde{\sigma}^{[\beta^2]} \beta^2,$$

where T_k are geometry-dependent switching functions, γ solvent macroscopic surface tension, β solvent Abraham's hydrogen bond basicity parameter, φ and ψ fraction of solvent atoms that are aromatic carbon and halogen atoms, respectively. Within the SMD model, the $\tilde{\sigma}_{Z_k}$, $\tilde{\sigma}_{Z_k Z_{k'}}$, $\tilde{\sigma}^{[\gamma]}, \tilde{\sigma}^{[\varphi^2]}, \tilde{\sigma}^{[\psi^2]}, \tilde{\sigma}^{[\beta^2]}$ parameters are fitted together with atomic radii for cavity construction.

Compensation of escaped charge from the cavity

Small amount of the solute charge escaped from the cavity gives rise to volume charge density $\beta(\mathbf{r})$ in the dielectric medium. [2,9,10] These charges contribute to polarization of the solute wavefunction, and their impact is especially important for anionic and zwitterionic structures - more diffuse wavefunctions and smaller cavities. Nevertheless, calculations with explicit volume charges would be demanding and techniques how to include the corresponding correction in a form of effective surface charges $\alpha(\mathbf{r})$ have been developed. One of these approaches is the electron density weighted correction. It is based on satisfaction of Gauss' law with the assumption that all solute charge density is accommodated in the cavity and is described by following equations:

$$\sigma^{corr}(\boldsymbol{s}) = \sigma(\boldsymbol{s}) + \alpha(\boldsymbol{s})$$

$$\alpha(\boldsymbol{s}) = \frac{\rho_{el}(\boldsymbol{s})}{\int_{\boldsymbol{\Omega}} \rho_{el}(\boldsymbol{s})d\boldsymbol{s}} Q_{exc}$$

$$Q_{exc} = -\frac{\epsilon - 1}{\epsilon} Q_{sol} - \int_{\boldsymbol{\Omega}} \sigma(\boldsymbol{s})$$

where σ(**r**) represents surface charges formed on the cavity surface by electric field of the solute and Q_{sol} is the total charge of the solute. Within another technique correction surface charges are added that generates the same electrostatic potential on the surface $\Phi^{\beta}(s)$ as the volume charges $\beta(\boldsymbol{r})$ i.e.

$$\hat{S}\alpha(\boldsymbol{s}) = \Phi^{\beta}(\boldsymbol{s}) \ ,$$

$$\hat{S}\sigma(\boldsymbol{s}) = \int_{\boldsymbol{\Omega}-\boldsymbol{\delta s}} \frac{\sigma(\boldsymbol{s}')}{|\boldsymbol{s} - \boldsymbol{s}'|} ds' + 1.07\, \sigma(\boldsymbol{s})\sqrt{4\pi a(\delta \boldsymbol{s})}$$

where the second term corresponds to the correction for the part of surface with area $a(\delta\boldsymbol{s})$ excluded from the integration. Eventually, this approach leads to the equations for the corrected charges σ^{corr}:

$$\left[\frac{\epsilon + 1}{\epsilon + 1}\hat{S} + \frac{1}{2\pi}\hat{S}\hat{D}^*\right]\sigma^{corr}(\boldsymbol{s}) = -\Phi^{\rho}(\boldsymbol{s}) + \frac{1}{2\pi}\hat{D}\Phi^{\rho}(\boldsymbol{s}) \ ,$$

$$\hat{D}\sigma(\boldsymbol{s}) = \int_{\boldsymbol{\Omega}-\boldsymbol{\delta s}} \sigma(\boldsymbol{s}') \frac{(\boldsymbol{s} - \boldsymbol{s}')\cdot \boldsymbol{n_s}}{|\boldsymbol{s} - \boldsymbol{s}'|^3} ds' \ ,$$

$$\hat{D}^*\sigma(\boldsymbol{s}) = -\int_{\boldsymbol{\Omega}-\boldsymbol{\delta s}} \sigma(\boldsymbol{s}') \frac{(\boldsymbol{s} - \boldsymbol{s}')\cdot \boldsymbol{n_s}}{|\boldsymbol{s} - \boldsymbol{s}'|^3} ds'$$

where $\Phi^{\rho}(s)$ is the potential of the solute and $\mathbf{n_s}$ is the outward normal vector to the cavity surface. The method is called effective charges method for D-PCM in the Gaussian code. A full description can be found in ref. [11]

Reference:

1. Tomasi, J.; Mennucci, B.; Cammi, R. Chem Rev 2005, 105(8), 2999-3093.
2. Klamt, A.; Jonas, V. J Chem Phys 1996, 105(22), 9972-9981.
3. Tomasi, J.; Cammi, R.; Mennucci, B.; Cappelli, C.; Corni, S. PCCP 2002, 4, 5697-5712.
4. Mennucci, B.; Cammi, R.; Tomasi, J. J Chem Phys 1998, 109(7), 2798-2807.
5. Floris, F. M.; Tomasi J.; Ahuir, L. P. J Comput Chem 1991, 12(7), 784-791.
6. Pierotti, R. A. Chem Rev 1976, 76, 717–726.
7. Cancès, E.; Mennucci, B. J Chem Phys 2001, 114, 4744-4745.
8. Marenich, A. V.; Cramer, C. J.; Truhlar, D. G. J Phys Chem B 2009, 113, 6378-6396.
9. Barone, V.; Cossi, M. Journal of Physical Chemistry A 1998, 102(11), 1995-2001.
10. Pye, C. C.; Ziegler, T. Theor Chem Acc 1999, 101, 396-401.
11. Cossi, M.; Rega, N.; Scalmani, G.; Barone, V. J Chem Phys 2001, 114, 5691-5701.

2. Calculated pK_a values for the best models in the 'direct' calculations, using the COSMOtherm program

Table S1: pK_w and pK_a values for all amino acids obtained within model *9* using all considered functionals.

	Ala		Arg			Asn		Asp			Cys			Glu		
Exp.	***2.3***	***9.7***	***2.0***	***9.0***	***12.1***	***2.2***	***8.7***	***2.0***	***3.7***	***9.7***	***1.9***	***8.1***	***10.3***	***2.2***	***4.2***	***9.6***
B3LYP	2.7	10.5	2.9	10.7	17.1	2.5	13.0	2.7	4.6	16.7	0.8	9.2	14.7	2.2	3.9	11.9
B3LYP-D	3.1	10.4	3.0	10.9	17.1	2.5	13.0	2.6	4.7	16.3	0.9	9.1	14.8	2.3	4.1	11.7
M11	1.8	9.5	1.0	11.0	14.7	1.6	12.0	1.2	3.4	14.2	0.4	6.9	13.9	0.2	2.9	10.9
M11-L	2.4	8.0	3.0	9.7	11.7	2.6	10.7	2.0	5.2	13.7	1.5	4.3	11.8	0.6	4.6	9.8
M06-2X	2.9	8.6	2.2	9.3	15.2	2.3	10.9	1.4	5.1	13.6	0.8	6.9	12.9	1.6	3.0	10.3
PBE0	2.3	10.7	2.4	10.8	16.6	2.9	13.1	2.4	4.2	15.8	0.7	7.5	15.1	1.4	3.9	11.6
revPBE0	2.4	10.6	2.4	10.7	16.7	2.9	13.1	2.3	4.2	15.7	0.7	7.7	14.9	1.4	3.9	11.4
TPSSh	2.0	11.0	1.8	11.3	16.5	2.2	13.2	2.1	4.1	16.1	0.4	8.2	15.3	1.4	3.5	12.4
ωB97X-D	3.3	10.3	3.2	11.0	17.0	2.4	12.9	3.1	4.3	15.0	2.0	4.1	14.5	1.6	3.8	11.6

	Gln		Gly		His			Ile		Leu		Lys			Met	
Exp.	***2.2***	***9.0***	***2.3***	***9.6***	***1.7***	***6.0***	***9.1***	***2.3***	***9.6***	***2.3***	***9.6***	***2.2***	***9.2***	***10.7***	***2.2***	***9.1***
B3LYP	2.2	9.5	2.3	10.4	3.4	5.5	9.7	1.6	10.3	0.2	10.9	0.4	9.2	10.6	1.3	8.7
B3LYP-D	2.6	9.9	2.3	10.4	4.2	6.2	10.3	1.2	10.7	0.4	10.7	-0.2	10.2	10.4	1.5	9.0
M11	1.0	8.9	1.0	9.7	2.4	4.7	9.1	-0.1	9.2	-1.3	10.3	-0.2	7.7	10.7	-0.6	7.6
M11-L	1.4	9.1	2.0	8.9	3.8	0.8	8.9	-0.2	8.6	-0.8	9.0	0.7	7.4	7.0	0.6	8.0
M06-2X	1.8	8.2	2.4	9.2	3.4	2.9	8.2	1.5	8.2	0.2	8.9	-0.8	7.8	9.1	0.8	6.7
PBE0	1.4	10.7	2.1	10.5	3.8	4.4	9.9	1.4	10.3	-0.4	10.7	0.1	9.7	10.0	0.4	8.7
revPBE0	1.4	10.7	2.0	10.5	3.8	4.5	9.9	1.4	10.2	-0.4	10.7	0.1	9.7	10.0	1.1	8.7
TPSSh	1.7	10.7	1.7	11.0	3.3	4.6	10.4	1.0	11.0	-0.5	11.8	-0.4	10.0	10.4	0.5	9.2
ωB97X-D	1.9	10.8	2.2	10.7	3.8	5.4	10.1	1.1	10.7	0.7	10.7	0.5	9.8	11.1	1.1	9.3

	Phe		Pro		Ser		Thr		Trp		Tyr			Val		pK_w
Exp.	***2.2***	***9.1***	***2.0***	***10.5***	***2.1***	***9.1***	***2.2***	***9.0***	***2.4***	***9.3***	***2.2***	***9.0***	***10.1***	***2.3***	***9.5***	***14.0***
B3LYP	2.0	11.5	0.0	14.4	2.4	12.4	2.3	12.2	2.4	11.1	2.9	11.8	15.2	2.1	11.1	15.0
B3LYP-D	1.9	10.6	-0.1	14.4	2.5	12.4	2.2	12.2	1.8	11.4	2.4	11.1	15.3	2.0	11.2	15.0
M11	0.8	10.3	-1.6	14.2	1.0	11.7	1.1	12.3	0.2	11.3	0.9	10.5	14.0	0.4	10.3	13.5
M11-L	0.4	9.7	-0.1	10.0	2.3	10.3	1.3	9.9	1.1	9.3	0.8	8.6	13.6	1.1	8.6	16.8
M06-2X	1.6	8.8	-0.7	12.4	2.0	10.8	1.7	11.7	1.5	10.0	1.4	9.9	14.8	1.5	9.3	16.0
PBE0	1.5	11.2	-0.4	14.0	2.3	12.3	1.9	12.7	1.7	11.7	1.3	11.4	14.8	1.3	11.2	15.8
revPBE0	1.4	11.1	-0.4	14.0	2.3	12.3	1.7	12.6	1.7	11.7	1.2	11.3	14.7	1.4	11.1	15.6
TPSSh	1.3	10.6	-0.5	14.6	2.0	12.7	1.3	13.1	2.2	11.9	0.7	11.8	14.5	1.2	11.6	14.9
ωB97X-D	2.3	11.2	-0.1	14.4	2.4	12.3	1.9	12.5	2.7	11.6	1.3	11.8	15.4	2.1	10.8	15.3

Table S2: pK_w and pK_a values for all amino acids obtained within model *10* using all considered functionals.

	Ala		Arg			Asn		Asp			Cys			Glu		
Exp.	***2.3***	***9.7***	***2.0***	***9.0***	***12.1***	***2.2***	***8.7***	***2.0***	***3.7***	***9.7***	***1.9***	***8.1***	***10.3***	***2.2***	***4.2***	***9.6***
B3LYP	4.1	9.9	1.6	7.3	21.5	3.7	11.7	4.3	5.6	14.6	2.3	10.8	14.2	3.7	4.9	10.8
B3LYP-D	4.4	9.7	1.6	7.3	21.7	3.7	11.5	4.1	5.6	14.7	2.3	10.8	14.3	3.7	5.1	10.5
M11	3.3	9.0	-0.5	7.7	19.6	2.6	11.0	2.8	4.3	12.8	1.9	8.9	13.6	1.8	4.1	10.2
M11-L	3.3	7.4	1.3	6.4	16.4	3.1	9.7	2.9	5.6	11.9	2.8	5.4	11.2	1.7	5.3	8.7
M06-2X	3.9	7.9	0.5	5.8	20.1	3.1	9.7	2.9	5.7	11.9	2.0	8.7	12.3	3.0	3.7	9.1
PBE0	3.6	10.2	1.1	7.4	21.4	4.0	11.8	4.0	5.2	14.5	2.4	9.3	14.5	3.1	4.9	10.6
revPBE0	3.6	10.0	1.2	7.2	21.5	4.0	11.8	3.9	5.2	14.4	2.4	9.4	14.5	3.0	4.9	10.4
TPSSh	3.6	10.4	0.7	7.9	21.4	3.6	12.1	3.9	5.1	15.0	2.2	9.9	15.0	3.1	4.6	11.6
ωB97X-D	4.6	9.7	1.7	7.6	22.1	3.5	11.7	4.5	5.4	12.9	3.2	10.1	14.1	3.2	4.8	10.6

	Gln		Gly		His			Ile		Leu		Lys			Met	
Exp.	***2.2***	***9.0***	***2.3***	***9.6***	***1.7***	***6.0***	***9.1***	***2.3***	***9.6***	***2.3***	***9.6***	***2.2***	***9.2***	***10.7***	***2.2***	***9.1***
B3LYP	3.3	8.4	3.5	9.6	2.3	9.8	9.3	2.6	9.6	1.3	10.3	1.9	7.7	11.5	2.5	7.6
B3LYP-D	3.6	8.7	3.6	9.6	2.6	10.0	9.3	2.3	9.8	1.4	10.1	0.9	9.0	11.1	2.7	7.8
M11	2.2	8.2	2.3	9.4	1.1	9.1	8.9	1.2	8.6	-0.2	9.8	-1.2	6.4	12.0	0.5	6.8
M11-L	2.4	8.1	2.7	8.0	2.2	5.2	8.3	0.5	7.8	-0.2	8.1	1.6	6.2	7.8	1.5	7.0
M06-2X	2.8	6.9	3.4	8.3	1.8	7.2	7.7	2.5	7.3	1.0	8.0	0.0	6.0	10.0	2.0	5.5
PBE0	2.8	9.6	3.2	9.8	2.8	8.5	9.5	2.4	9.7	0.6	10.2	1.5	8.2	10.9	1.7	7.8
revPBE0	2.8	9.6	3.1	9.8	2.8	8.5	9.4	2.4	9.7	0.6	10.2	1.5	8.2	10.9	2.4	7.7
TPSSh	3.1	9.7	2.8	10.5	2.4	8.7	10.2	2.0	10.5	0.7	11.3	1.1	8.6	11.4	1.8	8.4
ωB97X-D	3.2	9.7	3.4	10.0	2.4	9.7	9.6	2.6	9.6	1.7	10.2	1.8	8.4	12.2	2.3	8.3

	Phe		Pro		Ser		Thr		Trp		Tyr			Val		pK_w
Exp.	***2.2***	***9.1***	***2.0***	***10.5***	***2.1***	***9.1***	***2.2***	***9.0***	***2.4***	***9.3***	***2.2***	***9.0***	***10.1***	***2.3***	***9.5***	***14.0***
B3LYP	3.3	10.3	1.7	13.6	4.0	11.8	3.5	11.4	3.5	9.9	1.1	10.8	17.8	3.1	10.2	16.1
B3LYP-D	3.3	9.3	1.5	13.6	4.1	11.7	3.2	11.2	3.1	10.0	3.5	10.4	17.6	3.0	10.4	16.1
M11	2.0	9.4	0.1	13.8	2.6	11.4	2.1	11.8	1.4	10.4	0.6	9.9	16.4	1.7	9.6	14.7
M11-L	1.1	8.4	1.2	8.9	3.4	9.7	2.0	9.3	1.7	8.1	0.3	7.9	15.6	1.6	7.7	17.8
M06-2X	2.6	7.5	1.0	11.3	3.4	10.1	2.6	10.9	2.5	8.8	0.4	9.0	17.1	2.5	8.2	17.0
PBE0	2.9	9.9	1.5	13.1	4.0	11.7	3.0	12.1	3.1	10.3	0.9	10.7	17.2	2.4	10.4	16.9
revPBE0	2.7	9.9	1.4	13.1	4.0	11.7	2.9	11.8	3.1	10.4	4.2	14.1	20.6	2.5	10.3	16.7
TPSSh	2.8	9.7	1.5	13.8	3.7	12.3	2.5	12.6	3.4	10.8	0.8	11.2	17.0	2.4	11.0	15.9
ωB97X-D	3.1	9.3	1.7	13.6	4.0	11.9	3.0	12.0	4.1	10.2	1.0	11.1	17.9	3.2	10.0	16.4

Table S3: pK_w and pK_a values for all amino acids obtained within model *2* using all considered functionals.

	**Ala**		**Arg**			**Asn**		**Asp**			**Cys**			**Glu**		
Exp.	***2.3***	***9.7***	***2.0***	***9.0***	***12.1***	***2.2***	***8.7***	***2.0***	***3.7***	***9.7***	***1.9***	***8.1***	***10.3***	***2.2***	***4.2***	***9.6***
B3LYP	3.3	12.0	2.5	11.4	17.8	3.4	14.0	3.3	5.7	18.8	1.9	9.9	17.6	2.7	5.0	13.6
M11	2.3	10.7	0.4	11.6	15.0	2.3	12.7	1.9	4.1	15.9	1.7	7.6	16.4	0.8	3.7	12.4
M11-L	3.0	8.7	2.7	10.0	11.9	3.4	11.3	2.6	6.2	14.8	2.5	5.0	14.5	1.3	5.9	11.1
M06-2X	3.3	9.8	1.7	9.9	15.8	3.0	11.9	2.2	5.8	15.4	1.9	7.3	15.3	2.2	3.7	11.8
PBE0	2.8	12.0	2.0	11.5	17.3	3.6	14.1	3.0	5.2	17.8	1.8	8.1	17.7	1.9	4.8	13.3
revPBE0	2.8	11.9	2.0	11.4	17.4	3.6	14.1	2.9	5.1	17.8	1.8	8.2	17.6	1.8	4.8	13.2
TPSSh	2.6	12.3	1.3	11.9	17.2	3.0	14.2	2.8	5.0	18.2	1.4	8.8	18.1	1.9	4.5	14.1
ωB97X-D	3.9	11.7	2.9	11.8	17.7	3.3	13.9	3.9	5.5	17.1	3.1	8.8	17.3	2.4	4.9	13.5

	**Gln**		**Gly**		**His**			**Ile**		**Leu**		**Lys**			**Met**	
Exp.	***2.2***	***9.0***	***2.3***	***9.6***	***1.7***	***6.0***	***9.1***	***2.3***	***9.6***	***2.3***	***9.6***	***2.2***	***9.2***	***10.7***	***2.2***	***9.1***
B3LYP	2.9	11.2	3.1	11.7	3.6	6.9	12.0	2.5	11.9	1.5	12.7	1.3	11.6	10.0	2.6	11.1
M11	1.6	10.6	1.7	10.9	2.7	5.9	11.0	0.8	10.8	-0.2	11.7	0.7	9.6	10.1	0.7	9.8
M11-L	2.2	10.3	2.7	9.6	4.1	2.0	10.4	1.0	9.4	0.4	9.9	1.9	8.5	6.2	1.9	9.5
M06-2X	2.4	9.8	3.0	10.4	3.8	4.0	10.2	2.2	9.5	1.3	10.3	0.1	9.5	8.4	2.2	8.9
PBE0	2.2	12.4	2.7	11.7	4.0	5.7	12.0	2.2	11.7	0.7	12.6	0.9	11.7	9.5	1.6	11.1
revPBE0	2.2	12.4	2.6	11.7	3.9	5.8	12.0	2.2	11.6	0.7	12.5	0.9	11.7	9.5	2.4	11.1
TPSSh	2.2	12.5	2.3	12.3	3.6	5.9	12.5	1.8	12.3	0.7	13.5	0.5	11.9	9.9	1.8	11.5
ωB97X-D	2.6	12.7	3.0	12.0	4.3	6.6	12.2	2.3	12.0	1.9	12.5	1.7	11.7	10.8	2.6	11.5

	**Phe**		**Pro**		**Ser**		**Thr**		**Trp**		**Tyr**			**Val**		**pK_w**
Exp.	***2.2***	***9.1***	***2.0***	***10.5***	***2.1***	***9.1***	***2.2***	***9.0***	***2.4***	***9.3***	***2.2***	***9.0***	***10.1***	***2.3***	***9.5***	***14.0***
B3LYP	3.2	13.1	0.8	16.1	3.2	13.6	2.9	13.8	3.8	13.1	0.5	13.2	17.1	2.9	12.4	20.5
M11	1.9	11.7	-0.9	15.5	1.8	12.8	1.8	13.7	1.8	12.5	-1.2	12.0	15.5	1.2	11.3	18.5
M11-L	1.6	11.1	0.5	10.8	3.1	11.1	2.3	10.8	2.3	10.5	-0.2	9.7	14.8	2.0	9.1	22.0
M06-2X	2.9	10.2	0.0	13.7	2.8	11.8	2.5	12.8	2.7	11.6	-0.3	11.2	16.1	2.2	10.4	20.7
PBE0	2.7	12.7	0.3	15.5	3.0	13.5	2.3	14.2	3.2	13.3	0.0	12.9	16.4	2.2	12.4	21.0
revPBE0	2.6	12.7	0.2	15.5	3.0	13.4	2.2	14.1	3.1	13.3	-0.1	12.9	16.3	2.2	12.4	20.9
TPSSh	2.4	12.2	0.2	16.1	2.7	13.9	2.0	14.5	3.5	13.6	-0.3	13.4	16.2	2.0	12.8	20.2
ωB97X-D	3.8	12.6	0.7	16.1	3.3	13.5	2.8	14.0	4.4	13.2	0.6	13.2	17.2	2.9	12.2	20.8

Table S4: pK_w and pK_a values for all amino acids obtained with COSMO-RS calculations (model *16*) in the COSMOtherm program using all considered functionals.

	Ala		Arg			Asn		Asp			Cys			Glu		
Exp.	***2.3***	***9.7***	***2.0***	***9.0***	***12.1***	***2.2***	***8.7***	***2.0***	***3.7***	***9.7***	***1.9***	***8.1***	***10.3***	***2.2***	***4.2***	***9.6***
B3LYP	4.9	9.3	3.9	9.9	14.3	4.5	9.2	4.5	4.4	9.3	4.6	7.2	8.9	4.4	5.1	9.1
M11	3.7	8.7	2.8	9.1	13.5	3.3	8.5	3.3	3.6	7.9	3.6	5.7	8.0	3.5	3.7	8.6
M11-L	6.2	9.9	5.1	9.4	13.6	6.2	9.8	6.2	3.8	9.5	6.0	6.0	8.7	5.9	4.0	9.7
M06-2X	4.8	8.3	3.8	8.8	13.4	4.3	8.1	4.3	4.7	7.9	4.6	5.9	7.8	4.4	4.9	8.2
PBE0	5.4	9.9	4.4	10.5	14.8	5.2	9.9	5.2	4.9	10.0	5.2	7.1	9.5	5.0	5.7	9.9
revPBE0	5.3	9.8	4.2	10.4	14.8	5.0	9.9	5.1	4.8	10.0	5.1	7.1	9.4	4.9	5.6	9.8
TPSSh	5.5	10.6	4.5	11.1	14.9	5.3	10.6	5.3	5.0	10.8	5.3	7.9	10.1	5.0	6.0	10.3
ωB97X-D	5.8	10.2	5.0	10.8	15.3	5.4	10.1	5.4	5.3	10.1	5.5	7.8	9.7	5.4	5.9	10.0

	Gln		Gly		His			Ile		Leu		Lys			Met	
Exp.	***2.2***	***9.0***	***2.3***	***9.6***	***1.7***	***6.0***	***9.1***	***2.3***	***9.6***	***2.3***	***9.6***	***2.2***	***9.2***	***10.7***	***2.2***	***9.1***
B3LYP	4.4	9.3	4.5	9.1	4.6	8.9	9.2	4.3	9.3	4.0	10.3	4.7	10.0	10.8	4.2	9.4
M11	3.4	8.5	3.5	8.3	3.5	8.2	8.4	3.1	8.5	3.0	9.4	3.7	9.2	10.4	2.9	8.8
M11-L	5.9	10.1	6.0	9.7	5.6	8.4	9.7	5.3	9.7	5.3	10.5	6.2	9.6	10.6	5.8	10.0
M06-2X	4.4	8.3	4.6	8.0	4.7	7.3	8.1	4.1	8.3	4.0	9.1	4.3	8.8	10.0	4.3	8.4
PBE0	5.0	9.9	5.1	9.7	5.1	9.1	9.8	4.9	9.8	4.5	10.9	5.3	10.6	11.2	4.8	10.1
revPBE0	4.9	9.9	5.0	9.6	5.0	9.0	9.7	4.7	9.7	4.4	10.8	5.2	10.5	11.2	4.7	10.0
TPSSh	4.7	11.0	5.2	10.4	5.1	9.5	10.5	4.9	10.5	4.6	11.6	5.3	11.3	11.9	4.8	10.7
ωB97X-D	5.3	10.2	5.5	9.9	5.7	9.5	10.1	5.2	10.2	5.0	11.4	5.7	10.9	11.9	5.3	10.3

	Phe		Pro		Ser		Thr		Trp		Tyr			Val		pK_w
Exp.	***2.2***	***9.1***	***2.0***	***10.5***	***2.1***	***9.1***	***2.2***	***9.0***	***2.4***	***9.3***	***2.2***	***9.0***	***10.1***	***2.3***	***9.5***	***14.0***
B3LYP	4.4	9.6	3.6	11.4	4.3	9.3	3.8	9.4	4.4	9.9	4.5	9.4	11.0	4.4	9.2	13.3
M11	3.4	8.8	2.5	10.7	3.2	8.5	2.7	8.3	3.3	9.0	3.5	8.5	9.8	3.1	8.5	11.8
M11-L	5.9	10.0	5.2	10.8	5.9	9.8	5.2	9.9	6.0	10.2	6.0	8.9	11.6	5.5	9.3	15.4
M06-2X	4.3	8.6	3.5	10.2	4.3	8.2	3.7	8.0	4.2	8.8	4.4	8.3	10.9	4.2	8.2	13.8
PBE0	5.0	10.1	4.2	11.7	4.9	9.8	4.3	10.2	5.0	10.4	5.1	10.0	11.4	5.0	9.7	14.2
revPBE0	4.9	10.0	4.1	11.6	4.8	9.8	4.2	10.1	4.9	10.3	4.9	9.9	11.3	4.8	9.6	14.0
TPSSh	5.1	10.8	4.5	12.5	5.1	10.5	4.4	10.8	5.2	11.1	5.2	10.6	11.6	5.1	10.4	14.2
ωB97X-D	5.4	10.5	4.6	12.3	5.3	10.3	4.8	10.3	5.3	10.9	5.4	10.3	12.0	5.4	10.1	14.0

Table S5: Total RMSDs and RMSDs for specific pK_a values within selected solvent models. 'COOH' means all 1-carboxyl groups, 'NH_2' α-amino groups and 'side-chain' acidic or basic groups of side-chains of Arg, Asp, Cys, Glu, His, Lys, Tyr. For side-chain pK_a values another splitting is consider: 'side COOH' – carboxyl groups of Asp and Glu, 'side NH_2' – amino groups of Arg, His, and Lys, 'side SH' - thiol group of Cys, and 'side OH' – hydroxyl group of Tyr.

**Model**	**RMSDs**							
	**Total**	**COOH**	**NH_2**	**side-chain**	**side COOH**	**side NH_2**	**side OH**	**side SH**
	B3LYP							
2	3.36	1.02	4.29	3.97	3.53	3.34	7.00	1.79
8	2.13	**0.87**	1.90	4.29	1.85	5.08	6.54	1.37
9	2.23	0.95	2.80	2.99	2.33	2.88	5.07	1.03
10	2.51	1.18	2.09	5.11	2.68	5.85	7.67	2.62
16	1.60	2.22	**0.61**	1.52	0.85	2.08	0.94	0.95
	M11							
2	2.60	1.42	3.26	2.68	2.51	1.68	5.36	0.57
8	2.08	1.84	1.85	3.19	1.19	3.86	4.78	0.87
9	2.04	1.89	2.18	2.12	1.75	1.72	3.87	1.29
10	2.16	1.38	1.88	4.05	1.93	4.74	6.29	0.71
16	1.72	1.14	1.90	1.36	0.74	1.89	**0.30**	2.45
	M11-L							
2	2.34	1.12	1.92	3.79	3.98	3.51	4.72	3.14
8	1.90	1.34	1.50	3.35	3.70	2.75	4.40	3.01
9	2.21	1.54	2.33	3.16	1.93	3.00	4.44	3.95
10	1.89	1.07	1.62	3.44	2.96	3.02	5.49	2.78
16	2.47	3.64	0.73	1.46	**0.15**	1.64	1.51	2.12
	M06-2X							
2	2.37	1.08	2.45	3.37	3.09	2.79	6.03	0.88
8	2.11	1.21	2.15	3.57	1.46	4.10	5.80	0.75
9	1.74	1.24	1.67	2.79	2.16	2.69	4.70	1.19
10	2.18	1.04	1.90	4.23	2.34	4.67	6.98	0.56
16	1.63	2.14	1.11	**1.25**	0.86	**1.11**	0.81	2.28
	PBE0							
2	3.29	1.08	4.19	3.73	3.83	3.08	6.28	**0.05**
8	2.07	0.98	1.82	4.09	2.35	4.86	5.95	0.11
9	2.26	1.26	2.73	2.96	2.80	2.80	4.66	0.60
10	2.43	1.10	2.09	4.84	3.16	5.52	7.14	1.18
16	2.03	2.80	0.93	1.82	1.35	2.35	1.30	1.08
	revPBE0							
2	3.26	1.07	4.14	3.74	3.85	3.13	6.24	**0.06**
8	2.07	0.94	1.81	4.13	2.40	4.94	5.88	0.01
9	2.23	1.23	2.68	2.98	2.85	2.84	4.63	0.48
10	2.77	1.12	2.32	5.70	3.20	5.63	10.49	1.28
16	1.94	2.68	0.88	1.77	1.25	2.33	1.21	1.08
	TPSSh							
2	3.41	1.06	4.54	3.48	3.12	3.01	6.14	0.65
8	2.11	1.03	2.03	3.95	1.69	4.83	5.78	0.46
9	2.38	1.35	3.03	2.68	2.11	2.68	4.43	**0.06**
10	2.51	1.04	2.45	4.73	2.49	5.57	6.93	1.78
16	2.25	2.88	1.55	2.03	1.60	2.67	1.48	0.27
	ωB97X-D							
2	3.31	1.24	4.14	3.92	3.55	3.26	7.05	0.68
8	4.77	0.89	6.79	4.63	1.84	5.36	6.52	3.78
9	3.88	1.05	5.57	3.40	2.45	2.85	5.33	4.01
10	5.03	1.24	7.01	5.29	2.77	6.22	7.80	1.98
16	2.36	3.19	1.22	2.19	1.67	2.85	1.85	0.31

3. MSEs and RMSDs for selected models and specific pK_a values

Table S6: MSEs of pK_a values calculated using all regarded functionals and solvent models.

Model	B3LYP	M11	M11-L	M06-2X	PBE0	revPBE0	TPSSh	ωB97X-D
1	22.70	21.50	20.99	21.56	22.39	22.38	22.54	22.67
2	2.38	1.09	0.63	1.10	2.10	2.07	2.14	2.45
3	-8.24	-9.47	-9.66	-9.57	-8.43	-8.42	-8.53	-7.96
4	3.10	2.17	1.48	1.94	2.89	2.88	2.93	3.21
5	-7.70	-8.63	-8.95	-8.94	-7.81	-7.78	-7.91	-7.38
6	0.64	-0.67	-1.19	-0.69	0.46	0.42	0.59	1.22
7	1.09	-0.17	-0.57	-0.14	0.84	0.80	0.89	1.55
8	0.72	-0.49	-0.93	-0.49	0.55	0.53	0.67	1.17
9	1.16	0.01	-0.32	0.05	0.93	0.91	0.95	1.50
10	1.40	0.41	-0.25	0.19	1.27	1.47	1.42	2.10
11	3.17	2.12	1.35	1.88	2.98	3.19	3.09	3.20
12	1.82	0.88	0.34	0.71	1.65	1.85	1.70	2.41
13	13.09	12.22	10.79	11.95	12.61	12.60	12.77	12.93
14	16.76	15.90	14.55	15.66	16.37	16.36	16.53	16.66
15	15.32	14.55	13.20	14.30	14.94	14.93	15.10	15.25
16	1.05	0.37	1.33	0.72	1.29	1.25	1.43	1.42

pK_a values from the fitting

Table S7: RMSDs of pK_a values calculated using **Equation 8** when only parameter *b* is fitted. Data are presented for all regarded functionals and selected models. Lowest RMSDs for the individual functionals are in bold.

Model	B3LYP	M11	M11-L	M06-2X	PBE0	revPBE0	TPSSh	ωB97X-D
1	3.53	3.52	3.08	3.00	3.68	3.66	3.88	3.29
2	2.31	2.33	2.13	1.95	2.46	2.44	2.61	2.15
3	**1.74**	1.75	2.33	1.82	1.74	1.74	1.68	1.61
4	2.18	2.16	2.18	1.87	2.31	2.29	2.48	1.99
5	1.77	**1.66**	2.32	1.92	**1.73**	**1.73**	**1.62**	**1.60**
6	2.07	2.13	1.78	2.07	2.08	2.08	2.11	4.86
7	2.02	2.17	1.84	1.79	2.20	2.18	2.32	3.84
8	2.02	2.04	1.73	2.04	2.01	2.01	2.01	4.71
9	1.92	2.05	1.83	1.72	2.07	2.05	2.19	3.69
12	1.96	2.18	1.82	1.78	2.10	2.07	2.23	3.54
13	1.98	2.05	1.72	1.68	2.01	2.01	2.22	1.87
14	2.86	2.89	2.40	2.31	2.95	2.94	3.22	2.68
15	1.82	1.93	**1.70**	**1.42**	1.94	1.92	2.17	1.71

Table S8: pK_a values for all amino acids obtained by fitting parameter *b* in **Equation 8** and using difference in Gibbs free energy ΔG^0 between the protonated and deprotonated forms calculated within model *5* using all considered functionals. Parameter *a* is 0.73305 mol·kcal^{-1} and fitted parameter *b* in kcal·mol^{-1} is stated in the last column.

	**Ala**		**Arg**			**Asn**		**Asp**			**Cys**			**Glu**		
Exp.	***2.3***	***9.7***	***2.0***	***9.0***	***12.1***	***2.2***	***8.7***	***2.0***	***3.7***	***9.7***	***1.9***	***8.1***	***10.3***	***2.2***	***4.2***	***9.6***
B3LYP	3.0	7.4	2.7	6.8	13.8	2.4	10.4	2.7	4.5	14.5	1.4	7.9	13.7	2.1	5.9	8.3
M11	3.1	7.7	2.0	8.4	12.1	2.7	10.7	2.4	4.4	13.0	2.6	6.4	14.0	1.5	5.9	8.8
M11-L	3.1	5.4	3.3	6.7	9.5	3.6	8.9	2.8	5.4	11.7	2.7	3.0	12.0	1.3	6.9	7.1
M06-2X	4.0	6.8	3.1	6.7	13.3	3.7	9.5	2.8	5.9	12.4	2.7	6.6	13.0	3.0	6.1	8.0
PBE0	2.8	7.6	2.6	7.1	13.6	3.0	10.7	3.0	3.9	13.8	1.8	6.4	14.0	1.9	6.7	8.2
revPBE0	3.0	7.4	2.7	7.0	13.7	3.0	10.7	3.0	3.8	13.8	1.8	6.5	13.9	1.9	6.7	8.1
TPSSh	2.6	8.0	1.7	7.5	13.4	2.4	10.7	2.7	3.6	14.2	1.3	6.9	14.3	1.6	5.8	9.0
ωB97X-D	3.3	7.3	3.1	7.1	13.7	2.5	10.3	2.9	4.1	12.8	2.4	6.8	13.6	1.7	5.8	8.3

	**Gln**		**Gly**		**His**			**Ile**		**Leu**		**Lys**			**Met**	
Exp.	***2.2***	***9.0***	***2.3***	***9.6***	***1.7***	***6.0***	***9.1***	***2.3***	***9.6***	***2.3***	***9.6***	***2.2***	***9.2***	***10.7***	***2.2***	***9.1***
B3LYP	2.6	6.2	2.8	7.2	3.6	7.4	8.9	1.8	7.2	0.8	7.6	1.2	7.0	8.3	2.4	5.9
M11	2.2	6.9	2.5	7.7	3.3	7.8	9.4	1.0	7.6	0.3	8.1	1.3	6.6	9.7	1.4	6.0
M11-L	2.0	6.5	2.9	6.4	4.3	3.9	8.2	0.8	5.9	0.3	6.1	2.1	5.3	6.0	1.9	5.7
M06-2X	2.9	6.2	3.9	7.2	4.8	5.5	8.5	2.5	6.5	1.8	6.8	0.8	6.5	8.2	2.6	5.3
PBE0	1.9	7.4	2.8	7.4	4.5	6.4	9.0	2.1	7.3	0.4	7.9	1.1	7.4	8.2	1.7	6.2
revPBE0	1.9	7.6	2.7	7.5	4.4	6.5	9.0	2.1	7.3	0.4	7.8	1.1	7.4	8.3	2.2	6.2
TPSSh	1.9	7.8	2.4	8.1	3.9	6.6	9.6	1.5	7.9	0.2	8.6	0.6	7.6	8.6	1.8	6.7
ωB97X-D	2.0	7.7	2.5	7.6	4.0	6.7	9.1	1.3	7.5	1.1	7.6	1.4	7.3	9.1	2.2	6.4

	**Phe**		**Pro**		**Ser**		**Thr**		**Trp**		**Tyr**			**Val**		***b***
Exp.	***2.2***	***9.1***	***2.0***	***10.5***	***2.1***	***9.1***	***2.2***	***9.0***	***2.4***	***9.3***	***2.2***	***9.0***	***10.1***	***2.3***	***9.5***	***-272.7***
B3LYP	2.8	9.5	0.4	10.2	2.5	10.8	2.4	10.5	3.3	9.4	0.8	10.2	14.4	2.3	7.2	-267.9
M11	3.0	9.6	-0.1	11.0	2.2	11.3	2.2	11.9	2.2	10.4	0.7	10.3	14.0	1.6	7.6	-266.2
M11-L	1.6	8.8	0.8	6.0	3.1	9.6	2.2	10.1	2.1	8.2	1.0	7.8	12.7	1.9	4.9	-270.1
M06-2X	4.1	8.3	0.7	9.3	3.2	10.4	3.3	10.8	3.2	9.3	1.4	9.6	14.8	2.6	6.8	-266.5
PBE0	2.9	9.4	0.2	9.8	2.6	10.9	2.4	11.1	3.1	9.8	0.9	10.1	14.0	1.9	7.5	-268.9
revPBE0	2.9	9.4	0.2	9.9	2.6	10.9	2.2	11.1	3.0	9.9	0.8	10.1	13.9	1.9	7.5	-268.8
TPSSh	2.3	8.9	0.1	10.4	2.3	11.2	2.0	11.4	3.3	9.9	0.4	10.6	13.7	1.7	7.9	-269.7
ωB97X-D	3.3	9.1	0.1	10.1	2.3	10.8	1.9	10.6	3.7	9.7	1.0	10.2	14.3	2.1	7.1	-269.9

Table S9: RMSDs of pK_a values calculated using **Equation 8** when both parameters *a* and *b* are fitted. Data are collected for all regarded functionals and selected models.

Model	B3LYP	M11	M11-L	M06-2X	PBE0	revPBE0	TPSSh	ωB97X-D
1	1.11	1.08	1.97	1.31	1.13	1.13	1.06	1.08
2	1.09	1.03	2.34	1.30	1.13	1.13	1.02	1.05
3	1.52	1.45	2.86	1.76	1.52	1.52	1.35	1.43
4	1.16	1.04	2.58	1.39	1.17	1.17	1.05	1.09
5	1.63	1.47	3.02	1.89	1.59	1.59	1.38	1.50
6	1.41	1.30	1.65	1.65	1.35	1.36	1.21	2.42
7	1.04	1.00	1.54	1.27	1.07	1.07	0.94	1.89
8	1.47	1.33	1.61	1.70	1.40	1.41	1.26	2.44
9	1.08	1.02	1.53	1.30	1.10	1.09	0.97	1.91
12	1.13	1.07	**1.52**	1.39	1.13	1.13	0.99	1.92
13	0.92	0.74	2.54	1.13	0.88	0.89	0.79	0.87
14	0.88	0.82	2.33	1.08	0.90	0.91	0.82	0.83
15	**0.72**	**0.65**	2.58	**0.94**	**0.75**	**0.76**	**0.63**	**0.70**

Table S10: Fitted parameters *a* and *b* in **Equation 8** within model *15* for the individual functionals. They were used for determination of pK_a values in **Table S11**.

Functional	B3LYP	M11	M11-L	M06-2X	PBE0	revPBE0	TPSSh	ωB97X-D	***Exp.***
a ($mol \cdot kcal^{-1}$)	0.4988	0.4853	0.1976	0.5632	0.4877	0.4891	0.4634	0.5091	0.7331
b ($kcal \cdot mol^{-1}$)	-132.8	-128.4	-49.5	-150.0	-130.3	-130.7	-123.9	-136.9	-272.7

Table S11: pK_a values for all amino acids obtained by fitting both parameters *a* and *b* in **Equation 8** and using the difference in Gibbs free energy ΔG^0 between the protonated and deprotonated forms calculated within model *15* for all considered functionals. The calculated RMSDs for the given functionals are stated in the last column.

	Ala		Arg			Asn		Asp			Cys			Glu		
Exp.	***2.3***	***9.7***	***2.0***	***9.0***	***12.1***	***2.2***	***8.7***	***2.0***	***3.7***	***9.7***	***1.9***	***8.1***	***10.3***	***2.2***	***4.2***	***9.6***
B3LYP	2.8	9.3	1.7	8.4	13.3	2.6	9.0	2.5	4.0	10.8	1.8	8.5	10.1	2.4	6.0	9.1
M11	3.1	9.3	1.2	9.4	12.2	2.8	8.9	2.5	3.9	10.1	2.4	7.9	10.0	2.2	6.4	9.2
M11-L	4.9	6.8	4.7	6.9	7.7	4.9	6.9	4.6	5.6	7.4	4.8	5.8	7.0	4.5	6.5	6.8
M06-2X	3.5	8.9	1.6	8.5	13.9	2.9	8.6	2.3	4.8	10.3	2.1	8.2	9.9	2.5	6.7	9.1
PBE0	2.7	9.4	1.7	8.5	13.0	3.0	9.2	2.6	3.9	10.7	2.0	7.6	10.3	2.1	6.8	9.0
revPBE0	2.7	9.4	1.8	8.5	13.1	3.0	9.2	2.5	3.9	10.6	2.0	7.7	10.3	2.1	6.8	8.9
TPSSh	2.6	9.4	1.5	8.7	12.6	2.7	9.1	2.5	3.9	10.7	1.9	7.9	10.4	2.2	6.0	9.3
ωB97X-D	3.1	9.1	1.9	8.6	13.4	2.4	9.0	2.8	3.8	9.7	2.4	8.1	10.0	2.0	6.2	9.1

	Gln		Gly		His			Ile		Leu		Lys			Met	
Exp.	***2.2***	***9.0***	***2.3***	***9.6***	***1.7***	***6.0***	***9.1***	***2.3***	***9.6***	***2.3***	***9.6***	***2.2***	***9.2***	***10.7***	***2.2***	***9.1***
B3LYP	2.4	8.5	2.5	8.5	2.1	6.2	8.2	2.4	8.7	2.1	9.3	2.2	9.6	8.1	2.4	8.6
M11	2.5	8.6	2.5	8.9	1.8	6.4	8.3	2.0	8.9	1.9	9.5	2.6	9.1	8.9	2.0	8.6
M11-L	4.7	6.9	4.8	6.8	4.8	5.1	6.7	4.3	6.8	4.4	6.9	4.9	6.9	6.2	4.6	6.9
M06-2X	2.6	8.4	3.1	8.8	1.7	5.4	8.0	2.5	8.3	2.3	9.0	1.7	9.3	8.3	2.5	8.2
PBE0	2.3	9.1	2.6	8.8	2.5	5.6	8.3	2.3	8.7	1.8	9.4	2.2	9.8	8.0	1.9	8.7
revPBE0	2.3	9.1	2.5	8.8	2.5	5.6	8.3	2.3	8.7	1.8	9.3	2.2	9.8	8.0	2.4	8.7
TPSSh	2.1	9.3	2.4	8.9	2.4	5.7	8.5	2.4	8.9	1.9	9.7	2.1	9.7	8.1	2.2	8.8
ωB97X-D	2.1	9.3	2.4	8.8	1.6	6.2	8.3	1.9	8.9	2.2	9.2	2.3	9.8	8.6	2.2	8.9

	Phe		Pro		Ser		Thr		Trp		Tyr			Val		***RMSD***
Exp.	***2.2***	***9.1***	***2.0***	***10.5***	***2.1***	***9.1***	***2.2***	***9.0***	***2.4***	***9.3***	***2.2***	***9.0***	***10.1***	***2.3***	***9.5***	
B3LYP	2.5	8.9	1.8	11.1	2.4	8.4	2.0	8.8	2.7	8.9	1.2	8.9	11.8	2.8	9.2	0.72
M11	2.4	8.8	1.7	11.6	2.4	8.6	2.0	9.0	2.5	8.9	1.2	8.8	11.8	2.3	9.6	0.65
M11-L	4.3	7.0	4.5	7.0	4.8	6.6	4.4	6.5	4.6	6.7	4.2	6.5	8.0	4.6	6.8	2.58
M06-2X	2.8	7.9	1.7	11.0	2.6	8.3	2.1	8.7	2.8	8.6	1.3	8.6	13.2	2.7	9.1	0.94
PBE0	2.3	8.8	1.8	10.8	2.5	8.4	2.0	9.0	2.7	9.0	1.3	8.8	11.5	2.5	9.4	0.75
revPBE0	2.3	8.7	1.7	10.9	2.6	8.4	1.9	9.0	2.7	9.0	1.3	8.8	11.5	2.5	9.4	0.76
TPSSh	2.3	8.3	1.8	10.9	2.4	8.6	1.9	9.1	3.0	9.1	1.3	8.9	11.1	2.5	9.5	0.63
ωB97X-D	2.5	8.6	1.6	11.1	2.3	8.4	1.9	8.9	3.2	8.7	1.3	8.9	12.1	2.6	9.1	0.70
